# Downregulation of class II phosphoinositide 3-kinase PI3K-C2β delays cell division and potentiates the effect of docetaxel on cancer cell growth

**DOI:** 10.1186/s13046-019-1472-9

**Published:** 2019-11-21

**Authors:** Ouma Cisse, Muzthahid Quraishi, Federico Gulluni, Federica Guffanti, Ioanna Mavrommati, Methushaa Suthanthirakumaran, Lara C. R. Oh, Jessica N. Schlatter, Ambisha Sarvananthan, Massimo Broggini, Emilio Hirsch, Marco Falasca, Tania Maffucci

**Affiliations:** 1 Queen Mary University of London, Barts and The London School of Medicine and Dentistry, Blizard Institute, Centre for Cell Biology and Cutaneous Research, 4 Newark Street, London, E1 2AT UK; 2 Molecular Biotechnology Center, Department of Molecular Biotechnology and Health Sciences, University of Torino, Turin, Italy; 3 Laboratory of Molecular Pharmacology, Istituto di Ricerche Farmacologiche “Mario Negri IRCCS”, Milan, Italy; 4 Present address: The Institute of Cancer Research, London, UK; 5 Metabolic Signalling Group, School of Pharmacy and Biomedical Sciences, CHIRI Biosciences, Curtin University, Perth, Western Australia Australia

**Keywords:** Docetaxel, Mitosis, Phosphoinositide 3-kinase, PI3K-C2β, Prostate cancer

## Abstract

**Background:**

Alteration of signalling pathways regulating cell cycle progression is a common feature of cancer cells. Several drugs targeting distinct phases of the cell cycle have been developed but the inability of many of them to discriminate between normal and cancer cells has strongly limited their clinical potential because of their reduced efficacy at the concentrations used to limit adverse side effects. Mechanisms of resistance have also been described, further affecting their efficacy. Identification of novel targets that can potentiate the effect of these drugs or overcome drug resistance can provide a useful strategy to exploit the anti-cancer properties of these agents to their fullest.

**Methods:**

The class II PI3K isoform PI3K-C2β was downregulated in prostate cancer PC3 cells and cervical cancer HeLa cells using selective siRNAs and the effect on cell growth was determined in the absence or presence of the microtubule-stabilizing agent/anti-cancer drug docetaxel. Mitosis progression was monitored by time-lapse microscopy. Clonogenic assays were performed to determine the ability of PC3 and HeLa cells to form colonies upon PI3K-C2β downregulation in the absence or presence of docetaxel. Cell multi-nucleation was assessed by immunofluorescence. Tumour growth in vivo was assessed using a xenograft model of PC3 cells upon PI3K-C2β downregulation and in combination with docetaxel.

**Results:**

Downregulation of PI3K-C2β delays mitosis progression in PC3 and HeLa cells, resulting in reduced ability to form colonies in clonogenic assays in vitro. Compared to control cells, PC3 cells lacking PI3K-C2β form smaller and more compact colonies in vitro and they form tumours more slowly in vivo in the first weeks after cells implant. Stable and transient PI3K-C2β downregulation potentiates the effect of low concentrations of docetaxel on cancer cell growth. Combination of PI3K-C2β downregulation and docetaxel almost completely prevents colonies formation in clonogenic assays in vitro and strongly inhibits tumour growth in vivo.

**Conclusions:**

These data reveal a novel role for the class II PI3K PI3K-C2β during mitosis progression. Furthermore, data indicate that blockade of PI3K-C2β might represent a novel strategy to potentiate the effect of docetaxel on cancer cell growth.

## Background

The ability of cancer cells to sustain proliferative signals was the first of six hallmarks of cancer that were described in a seminal review attempting to rationalise the complexity of neoplastic disease [1]. More than a decade later, sustained proliferation was still considered as, arguably, the main feature of cancer cells [2]. Over the years, several studies have established that alteration of signalling pathways regulating cell cycle progression is a common strategy that many cancer cells exploit to sustain their proliferation [3–6]. As a result of these studies, the potential therapeutic value of targeting these pathways has been increasingly recognised [7, 8] and several compounds targeting distinct proteins involved in cell cycle regulation have been developed and proposed as potential anti-cancer drugs [5, 6]. Examples include inhibitors of cyclin dependent kinases (CDKs) [7–14] and indeed three highly selective inhibitors of CDK4/6 are now approved for clinical use in specific cancer settings [13, 14], with more CDKs inhibitors currently been tested in clinical trials [13]. Anti-mitotic drugs received considerable attention, especially following the early identification of the anti-cancer properties of microtubules-targeting agents, such as vinca alkaloids [15] and taxanes [16]. Indeed, drugs that impair microtubule dynamics have been used as frontline chemotherapeutics for several cancer types [17] and they are still amongst the classic chemotherapeutics used as primary treatment for many cancers [18], with new anti-microtubules agents also currently been tested in clinical trials [19]. Anti-microtubule agents, however, suffer from several limitations, such as their inefficacy towards many cancer types or towards responsive tumours that eventually develop mechanisms of resistance [18, 19]. In addition, these drugs are known to induce serious side effects due to inhibition of mitosis in other proliferating cells (mainly bone marrow and gut) as well as inhibition of other microtubules-dependent functions, such as neuronal processes [18, 19]. In an effort to overcome at least some of these limitations and to develop more selective anti-mitotic drugs, extensive investigation has been directed in the last years towards the identification of molecular targets, including kinases, motor proteins or multi-protein complexes that are specifically involved in mitosis and, possibly, are more specific for cancer cells. As a result, several new drugs targeting distinct proteins specifically required for mitosis progression have been designed [18–26], with many eventually progressing into clinical trials [19]. For most of them, however, the clinical impact has been far from ideal, as they have shown no improvement or indeed reduced efficacy compared to the classic microtubule-targeting agents [18, 24, 25]. Despite their limitations, the undeniable anti-cancer properties of anti-mitotic drugs is still driving a huge interest towards identification of novel compounds or specific drug combinations that might impact on cancer cells more specifically and efficiently [19].

Phosphoinositide 3-kinases (PI3Ks) are a family of lipid kinases that catalyse the phosphorylation of selective phosphoinositides at position 3 within their myo-inositol head groups [27–29]. Eight mammalian PI3K isoforms exist and they are divided into three classes mainly based on their substrate specificity [30, 31]. PI3Ks regulate a plethora of intracellular functions, including cell proliferation, survival, migration, intracellular trafficking and metabolism [32, 33]. Class I PI3Ks have a well-established role in regulation of cell cycle progression, mainly through synthesis of phosphatidylinositol 3,4,5-trisphosphate and activation of its downstream effector protein kinase B/Akt [34–36]. Specific roles during mitosis have also been described [37, 38], with evidence demonstrating the involvement of class I PI3Ks during mitotic entry, metaphase progression and spindle orientation [37]. Similarly, Akt inhibition has been reported to affect expression of Aurora A kinase, a key regulator of mitosis progression [39]. The only class III PI3K isoform, hVps34, also contributes to mitosis [40, 41], mainly by recruiting proteins required for the abscission step during cytokinesis through synthesis of its lipid product phosphatidylinositol 3-phosphate (PtdIns3*P*) [42, 43]. More recently, it has been demonstrated that the class II isoform PI3K-C2α is also required during mitotic progression, specifically during mitotic spindle formation [44]. Overall, these studies indicate that several members of the PI3K family contribute to cell cycle regulation and, more specifically, to mitosis progression, suggesting a complex and co-ordinated action of distinct PI3K isoforms during this cellular process. Interestingly, the observation that the involvement of PI3K-C2α in mitosis does not require its enzymatic activity [44] further suggests that the contribution of PI3K isoforms might go beyond the regulation of specific phosphoinositide pools.

Despite a study reporting activation of the class II isoform PI3K-C2β during G2/M transition in HL-60 cells [45], very little is known about the potential involvement of this enzyme during cancer cell cycle progression and mitosis in particular. In fact, while it is well established that PI3K-C2β is required for cancer cell migration and invasion [46–54], the involvement of this enzyme in cancer cell growth and proliferation is less clear. Here we report that downregulation of PI3K-C2β delays cancer cell division, resulting in reduced ability to form colonies in vitro and delayed tumour growth during the first weeks upon cells implant in vivo. Furthermore, downregulation of PI3K-C2β in combination with the microtubule-stabilizing agent docetaxel almost completely abolishes colonies formation in clonogenic assays in vitro and strongly inhibits tumour growth in vivo, suggesting that inhibition of PI3K-C2β can potentiate the effect of docetaxel on cancer cell growth.

## Methods

### Cell lines and transfections

PC3 and HeLa cells were maintained in Dulbecco’s modified Eagle’s medium supplemented with 10% (v/v) foetal bovine serum and 1% (v/v) penicillin/streptomycin (complete medium) and grown in a humidified incubator at 37 °C, 5% CO_2_ atmosphere. All reagents were from Thermo Fisher Scientific. Stable PC3 cell lines were generated as previously described [52]. Transient transfections of siRNAs were performed using Oligofectamine™ (Thermo Fisher Scientific, cat number: 12252–011) according to the manufacturer’s instructions and using the following siRNAs: PI3K-C2β (sequence 1): AAGAATGCGACGCCTGGCAAG (Qiagen); PI3K-C2β (sequence 2): ON-TARGETplus PIK3C2B siRNA cat number: J-006772-08 (Dharmacon); PI3K-C2β (sequence 3): ON-TARGETplus PIK3C2B siRNA cat number: J-006772-09 (Dharmacon); PI3K-C2α (sequence 1): AAGTCCAGTCACAGCGCAAAG (Qiagen); PI3K-C2α (sequence 2): AAGTACAGAATGAGGAGATGG (Qiagen); PI3K-C2α (sequence 3): ON-TARGETplus PIK3C2A siRNA cat number: J-006771-05 (Dharmacon); p110β: SMARTpool siGENOME PIK3CB siRNA cat number: M-003019-02 (Dharmacon). Non-targeting siRNA (Ambion) or ON-TARGETplus Non-targeting Pool (Dharmacon, cat number: D-001810-10) were used as control (si control). Additional control cells were treated with transfection reagent alone (oligo) or left non-transfected (NT).

### Cell growth and clonogenic assays

#### Cell counting

Stable cell lines were seeded in 12 well plates. Alternatively, cells, seeded in 12 well plates, were transfected as specified above. After 24 h, cells in complete medium were treated with docetaxel (Sigma Aldrich, cat number: 01885), or with the selective class I PI3K p110β inhibitor GSK2636771 (Generon Ltd., cat number: B2186) or the pan-PI3K inhibitor LY294002 (Cambridge Bioscience, cat number: CAY70290). Control cells were treated with vehicle alone (DMSO). Cells were manually counted at the indicated times using a Burker chamber and a light microscope at 10x magnification. All experiments were performed in duplicate.

#### Clonogenic assay

Stably or transiently transfected PC3 cells were plated in 6 well plates (200 cells/well) and incubated for 10 days in complete medium. HeLa cells were transfected, detached 24 h post transfection, plated in 6 well plates (100, 200 or 400 cells/well) and incubated for 7 days in complete medium. Where indicated, medium was supplemented with the indicated concentrations of docetaxel or DMSO. Colonies were then fixed with 4% paraformaldehyde (PFA) and stained with crystal violet (0.01% in PBS). Images were acquired using a bright field microscope. Alternatively, fixed colonies were incubated with HCS CellMask™ Deep Red (cat number: H32721, Thermo Fisher Scientific) and 4′,6-diamidino-2-phenylindole (DAPI, cat number: D1306, Thermo Fisher Scientific) and images were acquired and analysed using IN Cell Analyzer 2200 (GE Healthcare Life Sciences).

### Time-lapse microscopy

For data in Fig. 2a, PC3 cells were plated in 6 well plates and transfected with si control and distinct siRNAs targeting PI3K-C2β. After 48 h, cells were monitored for 19 h using a Zeiss Axiovert 200 M TimeLapse Epi-fluorescent Microscope combined to a CO_2_ and temperature controlled chamber (Solent Scientific). Images were acquired every 10–15 min using 20X 0.4NA LWD objective, QI imaging camera and MetaMorph software (Molecular Devices). Images were then stacked into movies using Image J. The time required by each cell to progress from rounding up to split into two cells and to complete separation of the two daughter cells was determined through frame-by-frame analysis of the recorded movies. For data in Fig. 2b, HeLa cells, plated on μ-Slide 8 Well (Ibidi), were imaged using a Leica TSC-II SP8 confocal microscope while incubated in a humidified chamber, at 37 °C and 5% CO_2_. Images were acquired every 10 min for 20 h, consistent with previous study [44].

### Cell cycle assay

PC3 cells were incubated in complete medium supplemented with 100 nM nocodazole for 24 h. After washing with PBS, cells that were still attached after nocodazole treatment were incubated in complete medium for further 2 h or 4 h. Cells were then washed once with PBS, detached and centrifuged at 1200 rpm for 5 min. Pelleted cells were fixed in ice-cold 70% ethanol, washed three times with PBS, centrifuged for 5 min and resuspended in 500 μl Vindellövs Propidium Iodide solution (50 μg/ml). Cells were analysed by flow cytometry collecting 20,000 events per sample using Fluorescence activated cell sorting (FACS) Diva software.

### Apoptosis assay

Assay was performed using FITC Annexin V Apoptosis Detection Kit with PI (Cambridge Bioscience, cat number: 640914) according to manufacturer’s instructions. Samples were analysed by flow cytometry collecting 20,000 events per sample using FACS Diva software.

### Western blotting analysis

Cells were washed and lysed with 2% SDS. Protein concentration was assessed using Pierce BCA Protein Assay Kit (Life Technologies Ltd. Invitrogen Division, cat number: 23227). Samples were separated by SDS-PAGE and transferred to nitrocellulose membranes. Membranes were incubated with 5% skimmed milk in PBS supplemented with 0.05% (v/v) Tween 20 (PBS-T) for 30 min at room temperature, followed by overnight incubation with primary antibodies at + 4 °C. Primary antibodies (and the corresponding dilutions, in PBS-T) were as follow: anti PI3K-C2β (BD Transduction laboratories, cat number: 611343, 1:500); anti PI3K-C2α (BD Transduction laboratories, cat number: 611046, 1:500); anti p110β (Cell Signaling Technology, cat number: 3011, 1:1000), anti α-Tubulin (Sigma Aldrich, cat number: T9026, 1:20,000); anti GAPDH (Cell Signaling Technology, cat number: 5174, 1:5000). After washing with PBS-T, membranes were incubated with secondary antibodies (Sigma Aldrich, peroxidase conjugate goat anti-rabbit IgG, cat number: A6154, peroxidase conjugate goat anti-mouse IgG, cat number: A0168, 1:10,000) for 1 h at room temperature, washed with PBS-T and exposed to ECL reagent (Merck™ Immobilon™ Western Chemiluminescent HRP Substrate, cat number: 11546345, Thermo Fisher Scientific).

### Immunofluorescence analysis

For immunofluorescence analysis, cells were seeded onto coverslips in 12 well plates. Where specified, cells were treated with the indicated concentrations of docetaxel or DMSO for 48 h or 72 h. Cells were then washed with PBS, fixed with 4% PFA and permeabilised with 0.25% Triton X-100 in PBS for 5 min. After washing with PBS, coverslips were incubated in PBS supplemented with 0.5% bovine serum albumin for 30 min, followed by incubation with anti α-Tubulin (Sigma Aldrich, cat number T9026, 1:1000) for 1 h at room temperature. Coverslips were then washed with PBS, incubated with secondary antibodies [Goat anti-mouse Alexa Fluor488, cat number: A-11001; Goat anti-mouse Alexa Fluor568, cat number: A-11004, all from Thermo Fisher Scientific] for 1 h, washed with PBS and incubated with DAPI (1:1000) for 5 min. Images were acquired using a Leica DM4000 microscope and the MetaMorph® Microscopy Automation and Image Analysis Software. Image J was used for cell counting and image analysis. For experiments in Fig. 6, Additional file 8: Figure S7 and Additional file 11: Figure S10, the number of cells containing one nucleus, two nuclei or three nuclei or more as well as the total number of cells were determined in each image.

### In vivo experiments

Nude immunodeficient male mice were obtained from Envigo-Italy and maintained under specific pathogen-free conditions with food and water provided ad libitum. Procedures involving animals and their care were conducted in conformity with institutional guidelines that are in compliance with National Governing Law (D. lg 26/2014; Authorization no.19/2008-A issued March 6, 2008 by Ministry of Health, Italy) and International EU directive and guidelines (EEC Council Directive 2010/63/UE). The Statement of Compliance (Assurance) with the Public Health Service (PHS) Policy on Human Care and Use of Laboratory Animals was recently reviewed (9/9/2014) and will expire on September 30, 2019 (Animal Welfare Assurance #A5023–01). PC3 cells expressing (sh scrambled, clone 3) or lacking (sh PI3K-C2β, clone 3) PI3K-C2β were injected subcutaneously into the flanks of nude mice (*n* = 7 mice/group). After tumours reached approximately 150 mm^3^ in size, mice were randomised and treated with 3 mg/Kg docetaxel (IV) or corresponding vehicle. Tumour diameters were measured with a caliper twice weekly until animals were sacrificed. Body weights were measured twice weekly. When required, mice were humanely sacrificed via a rising concentration of CO_2_ to near 100% followed by cervical dislocation. Statistical analysis was performed by multiple T-test using the Holm-Sidak method, with alpha = 0.05. T/C (%) values were measured at the indicated days using the formula: weights of tumours from docetaxel-treated (T) mice/weights of tumours from vehicle-treated (C) X100.

## Results

### Downregulation of PI3K-C2β reduces 2D colonies formation from single PC3 cells

Several studies have established a role for the class II PI3K isoform PI3K-C2β in regulation of cell migration [46–53] as well as cancer cell invasion [50, 52] and experimental metastases models [50, 54]. On the other hand, the specific contribution of the enzyme to cancer cell growth and proliferation is, in general, less clear. For instance, studies from our and other laboratories demonstrated that downregulation of PI3K-C2β specifically reduced anchorage-independent growth of breast cancer [50] and neuroblastoma [55] cells without affecting growth of cells in normal growing conditions.

**Fig. 1:**
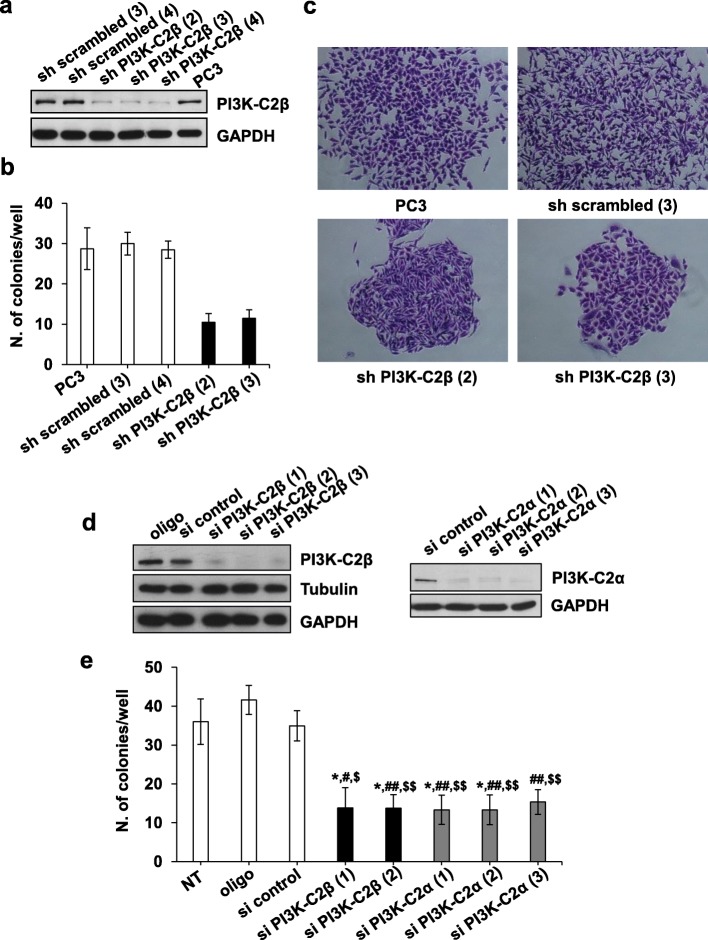
Stable and transient PI3K-C2β downregulation inhibits 2D colonies formation. **a** Representative blot confirming downregulation of PI3K-C2β in all stable PC3 clones lacking PI3K-C2β (shPI3K-C2β) that were used in this study. Levels of PI3K-C2β in the corresponding control stable clones (sh scrambled) compared to parental PC3 cells are also shown. GAPDH was used as loading control. **b, c** PC3 cells and the indicated stable clones were plated as single cells in 6 well plates (200 cells/well) and incubated in complete media for 10 days. Cells were then fixed and stained with crystal violet, images were collected and 2D colonies were manually counted. Data in (**b**) indicate the number of colonies/well and are means ± s.d. of *n* = 2 independent experiments (PC3, *n* = 4). Representative images of colonies stained with crystal violet at the end of the experiment are shown in (**c**). **d** PC3 cells were transfected with siRNAs specifically targeting PI3K-C2β or PI3K-C2α. Control cells were transfected with a non-targeting siRNA (si control) or transfection reagent alone (oligo). Representative blots confirming efficient downregulation of the enzymes by all siRNAs used in this study. Tubulin and GAPDH were used as loading controls. **e** PC3 cells were transfected as in (**d**). Non transfected cells (NT) were also used as an additional control. After 24 h, cells were detached and plated as described in (**b, c**). Data indicate the number of colonies/well and are means ± s.e.m. of *n* ≥ 3 independent experiments. *p < 0.05 vs NT; #*p* < 0.05, ^##^*p* < 0.01 vs oligo; ^$^*p* < 0.05, ^$$^*p* < 0.01 vs si control (two-tailed, unpaired t-Test with Welch’s correction)

We recently reported that downregulation of PI3K-C2β reduced migration and invasion of PC3 prostate cancer cells [52]. In this study, growth of stable cell lines up to 96 h did not appear to differ whether cells expressed or lacked PI3K-C2β. Similarly, transient downregulation of the enzyme using a selective siRNA did not seem to reduce the number of PC3 cells assessed at 72 h post transfection [52]. As these experiments were only performed in normal growing conditions and up to 72-96 h, we decided to investigate the potential effect of PI3K-C2β downregulation on PC3 growth in more detail. Specifically, we performed clonogenic assays to determine whether downregulation of the enzyme affected the ability of PC3 to form colonies when plated as single cells in 6 well plates and incubated in complete medium for 10 days (2D colonies). First, experiments were performed using stable clonal cell lines previously generated in our laboratory upon transfection with a selective PI3K-C2β-targeting shRNA (sh PI3K-C2β cells) or the corresponding, non targeting shRNA (sh scrambled cells) [52]. Downregulation of the enzyme in all clones used in this study was confirmed by Western blot (Fig. 1a). Interestingly, we observed that sh PI3K-C2β cells formed fewer 2D colonies (defined as groups of >50–65 cells) than sh scrambled cells or parental cells (Fig. 1b). Additionally, cells lacking PI3K-C2β formed more compact and less spread 2D colonies compared to control cells (Fig. 1c, Additional file 2: Figure S1). To rule out the possibility that the effect was due to potential adaptation of the stable cell lines to the chronic absence of PI3K-C2β, clonogenic assays were repeated using PC3 cells transiently transfected with siRNAs targeting PI3K-C2β. Cells were also transfected with siRNAs targeting another class II PI3K isoform, PI3K-C2α, in order to determine whether the effects were specific for PI3K-C2β. Downregulation of both enzymes was detectable already within 24 h from transfection and was still efficient at 72 h post-transfection (Fig. 1d). Data showed that transient downregulation of PI3K-C2β using two distinct siRNAs resulted in significant reduction of 2D colonies number compared to non-transfected cells (NT), cells treated with transfection reagent alone (oligo) or transfected with a non-targeting (si control) siRNA (Fig. 1e, Additional file 3: Figure S2). Downregulation of PI3K-C2α also reduced colonies number (Fig. 1e; Additional file 3: Figure S2).

To investigate this further, experiments were repeated and colonies were analysed using IN Cell Analyzer 2200. Data confirmed that stable cell lines lacking PI3K-C2β formed significantly fewer 2D colonies (containing ≥50 cells) compared to control cells (Additional file 4: Figure S3a). Importantly, sh PI3K-C2β cells formed a higher number of smaller cell aggregates (containing <50 cells) compared to control cells (Additional file 4: Figure S3b), indicating that downregulation of PI3K-C2β did not block the overall ability of single PC3 cells to divide and it did not induce cell death. The latter conclusion was consistent with our previous study reporting that transient downregulation of PI3K-C2β did not induce apoptosis in PC3 cells [52], which was confirmed by additional data in the stable cell lines (Additional file 4: Figure S3c). Downregulation of PI3K-C2α, on the other hand, slightly increased the percentage of apoptotic cells assessed by Annexin V/FACS analysis (Additional file 4: Figure S3d), which is consistent with previous studies [49, 56] and suggests that the two class II enzymes regulate distinct cellular functions in PC3 cells.

Taken together, these data indicate that downregulation of PI3K-C2β reduces the ability of PC3 to form colonies in vitro, possibly due to delayed cell proliferation.

### Downregulation of PI3K-C2β delays cancer cell division

**Fig. 2:**
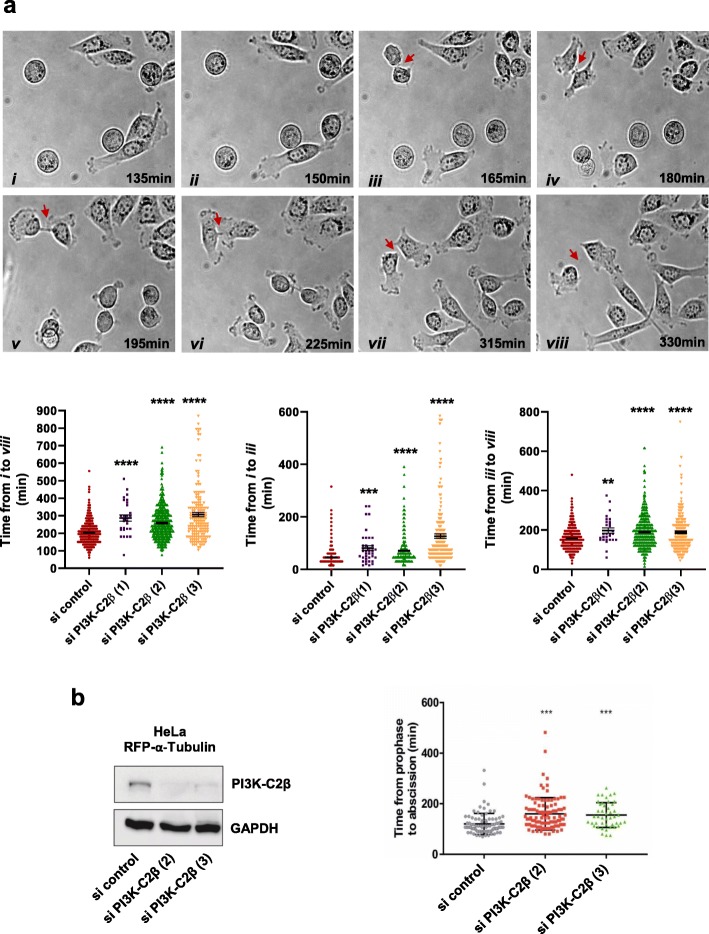
PI3K-C2β downregulation delays cell division. **a** PC3 cells were transfected with the indicated siRNAs. After 48 h, cells were monitored by time-lapse microscopy for 19 h. Representative images of si control-transfected PC3 cells acquired at the indicated minutes are shown. Arrows indicate the intercellular bridges. Graphs indicate the time required by each cell to progress from cell rounding to complete separation of daughter cells (from *i* to *viii*), from cell rounding to splitting into two cells (from *i* to *iii*) and from the appearance of the two daughter cells to their complete separation (from *iii* to *viii*). Data are from *n* = 3 [si control, si PI3K-C2β (2)], *n* = 2 [si PI3K-C2β (1)] and *n* = 2 [si PI3K-C2β (3)] independent experiments performed in duplicate. The total numbers of cells analysed were as follows: 598 (si control), 29 [si PI3K-C2β (1], 447 [si PI3K-C2β (2)] and 161 [si PI3K-C2β (3)]. **p < 0.01, ***p < 0.001, ****p < 0.0001 vs si control (two tailed, unpaired t-Test with Welch’s correction). **b** HeLa cells overexpressing RFP-α-tubulin were transfected with the indicated siRNAs. Efficient downregulation of PI3K-C2β was confirmed by Western blotting. GAPDH was used as loading control. After 24 h, cells were monitored by time-lapse microscopy for further 20 h. Graphs indicate the time required by each cell to progress from prophase to abscission. ***p < 0.001

To investigate the possibility that PI3K-C2β downregulation might affect the rate of cell proliferation, stable cells expressing (sh scrambled) and lacking (sh PI3K-C2β) the enzyme were treated with nocodazole for 24 h. After washing with PBS, cells that were still attached were incubated in complete medium for further 2 h or 4 h. Cell cycle analysis indicated that nocodazole treatment increased the percentage of cells in G2/M phase in both cell lines (sh scrambled: 45.9 ± 5.1, sh PI3K-C2β: 45.2 ± 4.6 compared to 19.6 ± 1.6 and 17.4 ± 1.2 in cells left in complete medium without nocodazole for the whole duration of the experiment). Upon nocodazole removal and in the analysed timeframe, we observed that a higher percentage of sh PI3K-C2β cells remained in the G2/M phases compared to control, sh scrambled cells (Additional file 5: Figure S4). Consistently, the percentage of sh PI3K-C2β cells in G1 phase of the cell cycle was reduced compared to sh scrambled cells (Additional file 5: Figure S4). These data indicated that progression from G2/M to G1 upon nocodazole removal was delayed in cells lacking PI3K-C2β, suggesting a potential role for the enzyme during cell mitosis/division. To investigate this possibility further, PC3 cells were transfected with siRNAs targeting PI3K-C2β and a control, non targeting siRNA and monitored by time-lapse microscopy to assess the time required by each cell to divide. This analysis revealed that downregulation of PI3K-C2β significantly increased the time required by the cells to progress from cell rounding (Fig. 2a, *i*) to complete breakdown of intercellular bridge and separation of the two daughter cells (abscission, Fig. 2a, *viii*). More specifically, PI3K-C2β downregulation appeared to delay progression from cell rounding to formation of the two daughter cells linked by the intercellular bridge (Fig. 2a, *i* to *iii*) and the time required to progress from this latter stage to complete abscission (Fig. 2a, *iii* to *viii*). Taken together, these data indicate that PI3K-C2β downregulation delays mitosis progression in PC3 cells.

As we previously reported that transient PI3K-C2β downregulation did not reduce PC3 cell numbers assessed at 72 h post-transfection [52], we decided to investigate whether the delayed cell mitosis detected in cells upon transient PI3K-C2β downregulation might require a longer time to be able to affect cell growth. Indeed, we observed that downregulation of the enzyme did eventually reduce the numbers of PC3 cells when measured at 120 h post-transfection (Additional file 6: Figure S5a). We next compared the effect of PI3K-C2β downregulation to modulation of other PI3K isoforms that have been involved in regulation of PC3 cell growth/viability. First, we determined the effect of modulation of the class I PI3K p110β, whose role in regulation of PC3 cell growth has been previously reported [57, 58]. Our data showed that both downregulation (Additional file 6: Figure S5b) and selective chemical inhibition (Additional file 6: Figure S5c) of p110β reduced PC3 cell numbers in a much shorter timeframe compared to PI3K-C2β downregulation, with the effect already detectable within 72 h from incubation with the inhibitor or siRNA transfection. Furthermore, inhibition of p110β resulted in increased percentage of cells in the G1 phase of the cell cycle at 72 h (Additional file 1: Table S1), which was not detected in these cells upon PI3K-C2β downregulation in the same timeframe [52]. We further observed that PI3K-C2α downregulation was also able to reduce PC3 cell numbers in a shorter timeframe compared to PI3K-C2β downregulation, with the effect detectable within 72 h and 96 h from transfection (Additional file 6: Figure S5d), consistent with our previous data indicating that PI3K-C2α (Additional file 4: Figure S3d) but not PI3K-C2β [52] is involved in PC3 cell survival/apoptosis. Interestingly, chemical inhibition of p110β in combination with PI3K-C2α downregulation further reduced cells number compared to each treatment alone (Additional file 6: Figure S5e), possibly supporting the hypothesis that p110β and PI3K-C2α affect distinct signalling pathways involved in cell viability/growth. Taken together these data indicate that downregulation of PI3K-C2β delays PC3 cell mitosis, eventually resulting in delayed cell growth through distinct cellular mechanisms compared to other PI3K isoforms.

Previous studies have demonstrated that the phosphoinositide PtdIns3*P* is involved in the recruitment of proteins crucial for cytokinesis to the midbody [42, 43]. Interestingly, we reported that PI3K-C2β regulates the synthesis of a pool of PtdIns3*P* in cervical cancer HeLa cells [46]. Whether PI3K-C2β, possibly through PtdIns3*P*, contributed to mitosis progression/cell proliferation in these cells was not investigated in our previous study [46]. To investigate further the involvement of the enzyme in mitosis progression, we therefore performed additional time-lapse microscopy analyses in these cells. Specifically, HeLa cells stably expressing RFP-α-tubulin were used in these experiments as visualisation of fluorescently-labelled microtubules remodelling allowed a more precise analysis of the effect of PI3K-C2β downregulation on the distinct phases of cell mitosis. Cells were transfected with siRNAs targeting PI3K-C2β or a non-targeting siRNA (Fig. 2b) and monitored for 20 h. Single cell analysis revealed that PI3K-C2β downregulation with two distinct siRNAs increased the time required to progress from prophase to abscission (Fig. 2b). Consistent with the delayed cell mitosis, PI3K-C2β downregulation reduced the numbers of HeLa cells (Additional file 6: Figure S5f).

Taken together, these data indicate that downregulation of PI3K-C2β increases the time required for cell division, revealing a novel role for the enzyme during mitosis progression.

### Downregulation of PI3K-C2β potentiates the effect of docetaxel in vitro

As data so far indicated that PI3K-C2β was involved in mitosis progression, we decided to determine the effect of downregulation of the enzyme in combination with docetaxel, a drug belonging to the family of taxanes that affect cell mitosis by binding to the β subunit of tubulin therefore impairing microtubules dynamics [59]. It was reported originally that the mechanisms of action of taxanes might be concentration-dependent [60], with studies further indicating that low concentrations of taxanes specifically affect mitotic progression by altering mitotic spindle microtubule dynamics [61]. Consistent with this, we observed that treatment of PC3 cells with low concentration of docetaxel (0.5 nM) for 72 h reduced cell numbers (Additional file 7: Figure S6a) but increased the percentage of apoptotic cells only slightly (Additional file 7: Figure S6b) whereas a clear increase in the percentage of apoptotic cells was detected using higher concentrations of the drug (Additional file 7: Figure S6b). On the other hand, treatment with 0.5 nM docetaxel increased the percentage of multi-nucleated PC3 cells (Additional file 7: Figure S6c), as previously reported [60, 61]. Similarly, treatment with low concentrations of docetaxel for 72 h induced multi-nucleation in HeLa cells strongly (Additional file 8: Figure S7a, b). Multi-nucleation was already detectable after 48 h and no major differences were detected between the percentage of multi-nucleated HeLa cells in cells treated with transfection reagent (oligo) or transfected with a control siRNA (Additional file 8: Figure S7c).

**Fig. 3:**
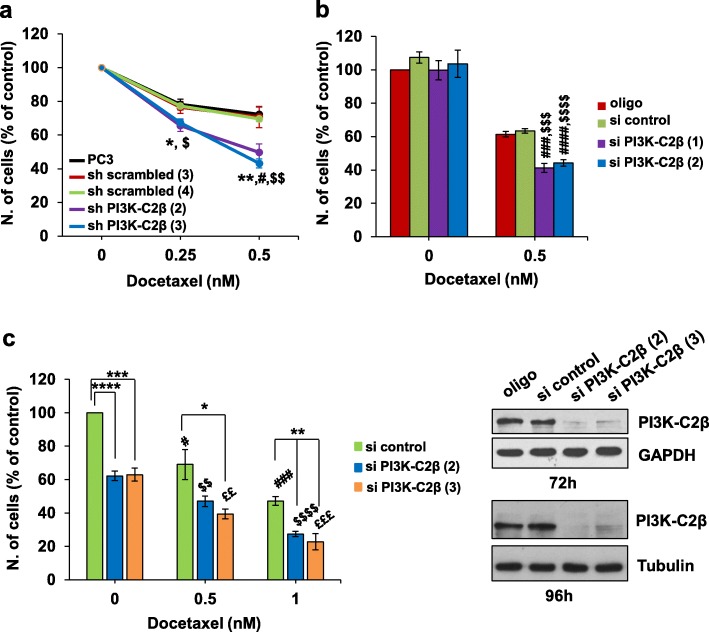
PI3K-C2β downregulation potentiates the effect of docetaxel on cell numbers. **a** PC3 cells and the indicated stable cell lines were incubated with the indicated concentrations of docetaxel for 72 h before cell counting. For each cell line data are expressed as percentage of cells treated with DMSO (control) and are means ± s.e.m. of: *n* = 3–10 (PC3), *n* = 3–4 (sh scrambled clone 3), *n* = 3 (sh scrambled clone 4, sh PI3K-C2β clone 4), and *n* = 6–8 (sh PI3K-C2β clone 3) independent experiments performed in duplicate. No statistical significant difference was detected in the number of cells treated with DMSO between the different cell lines. For both sh PI3K-C2β clones: *p < 0.05, **p < 0.01 vs corresponding PC3; ^#^*p* < 0.05 vs corresponding sh scrambled (3); ^$^*p* < 0.05, ^$$^*p* < 0.01 vs corresponding sh scrambled (4) (two tailed, unpaired t-Test with Welch’s correction). **b** PC3 cells were transfected with the indicated siRNAs. After 24 h, cells were incubated in complete media supplemented with 0.5 nM docetaxel or DMSO for further 72 h. The number of cells was assessed by cell counting. Data are expressed as percentage of cells transfected with transfection reagent and treated with DMSO (control) and are means ± s.e.m. of *n* = 6 independent experiments performed in duplicate. ^###^*p* < 0.001, ^####^*p* < 0.0001 vs corresponding oligo; ^$$$^*p* < 0.001, ^$$$$^*p* < 0.0001 vs corresponding si control (two tailed, unpaired t-Test with Welch’s correction). **c** HeLa cells were transfected with the indicated siRNAs. After 24 h, cells were incubated in complete media supplemented with the indicated concentrations of docetaxel or DMSO for further 72 h. Data indicate number of cells assessed by cell counting and are expressed as percentage of cells transfected with si control and treated with DMSO (control). Data are means ± s.e.m. of *n* = 4–5 independent experiments performed in duplicate. *p<0.05 **p < 0.01, ***p < 0.001, ****p < 0.0001 vs corresponding si control; ^#^*p* < 0.05, ^###^*p* < 0.001 vs si control/DMSO; ^$$^*p* < 0.01, ^$$$$^*p* < 0.0001 vs si PI3K-C2β (2)/DMSO; ^££^*p* < 0.01, ^£££^*p* < 0.0001 vs si PI3K-C2β (3)/DMSO (two tailed, unpaired t-Test with Welch’s correction). Downregulation of the enzyme at the indicated times was confirmed by Western blotting. Tubulin and GAPDH were used as loading control

We then investigated the effect of low concentrations of docetaxel on parental PC3 and on the stable sh scrambled and sh PI3K-C2β cell lines. Treatment of all cell lines with increasing concentrations of docetaxel significantly reduced the number of cells assessed at 72 h (Fig. 3a). Importantly, docetaxel reduced cells number more potently in cells lacking PI3K-C2β compared to all cell lines expressing the enzyme, especially when used at a concentration of 0.5 nM (Fig. 3a). We next investigated the effect of low concentrations of docetaxel on PC3 cells transiently transfected with PI3K-C2β-targeting siRNAs. Notably, in these experiments, counting was performed at 96 h post-transfection, i.e. at a time point when downregulation of PI3K-C2β per se was not able to affect cell growth yet. Indeed, no difference was detected between control cells and cells transfected with the PI3K-C2β-targeting siRNAs in the absence of docetaxel (Fig. 3b). On the other hand, we observed that downregulation of the enzyme potentiated the effect of docetaxel, with the drug reducing the number of cells more potently in cells lacking PI3K-C2β compared to control cells (Fig. 3b), consistent with data obtained in the stable cell lines. In parallel experiments, downregulation of PI3K-C2α reduced the number of cells to the same extent as treatment with 0.5 nM docetaxel in cells expressing the enzyme (Additional file 9: Figure S8a), further suggesting that the two class II PI3K isoforms affect cell growth through distinct intracellular mechanisms. Docetaxel treatment further reduced number of PI3K-C2α knockdown cells (Additional file 9: Figure S8a).

We next determined the effect of docetaxel in HeLa cells upon transient downregulation of PI3K-C2β. Consistent with our previous data, downregulation of PI3K-C2β per se reduced the number of HeLa cells (Fig. 3c). Treatment with low concentrations of docetaxel reduced the number of all cells in a dose-dependent manner (Fig. 3c). Importantly, combination of PI3K-C2β downregulation and low concentrations of docetaxel reduced the number of cells more potently than downregulation of the enzyme alone or treatment with the same concentration of the drug in control cells (Fig. 3c).

**Fig. 4:**
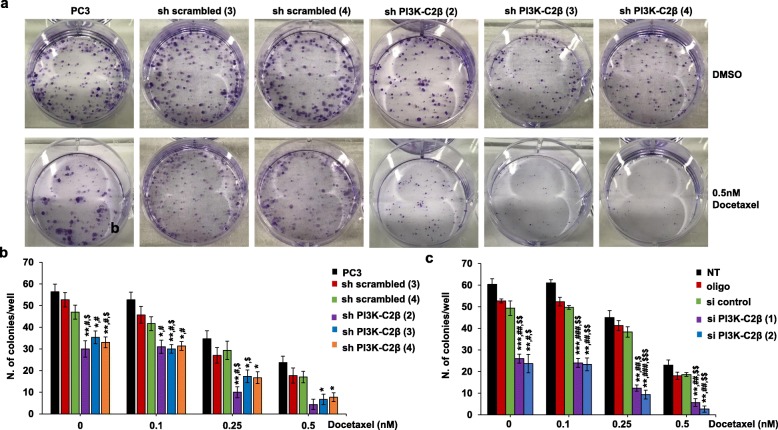
Combination of PI3K-C2β downregulation and docetaxel treatment strongly inhibits 2D colonies of PC3 cells in clonogenic assays. PC3 cells and stable cell lines (**a, b**) were plated as single cells in 6 well plates (200 cells/well). Alternatively, PC3 were transfected with the indicated siRNAs or treated with transfection reagent alone (oligo). Non-transfected (NT) cells were also used as additional control cells. After 48 h, cells were detached and plated as single cells (**c**). Cells were incubated in complete media for 10 days in the presence of the indicated concentrations of docetaxel (or vehicle, DMSO) before being fixed and stained with crystal violet. Representative images of 2D colonies from PC3 and stable cell lines at the end of the experiment are shown in (**a**). Data in (**b**) and (**c**) indicate the number of colonies/well (>65 cells) and are means ± s.e.m. of *n* = 3 independent experiments performed in duplicate. In (**b**): *p < 0.05, **p < 0.01 vs corresponding PC3; ^#^*p* < 0.05 vs corresponding sh scrambled (3); ^$^*p* < 0.05 vs corresponding sh scrambled (4) (two tailed, unpaired t-Test with Welch’s correction). In (**c**): **p < 0.01, ***p < 0.001 vs corresponding NT; ^#^*p* < 0.05, ^##^*p* < 0.01, ^###^*p* < 0.001 vs corresponding oligo; ^$^*p* < 0.05, ^$$^*p* < 0.01, ^$$$^*p* < 0.001 vs correspondent si control (two tailed, unpaired t-Test with Welch’s correction)

**Fig. 5:**
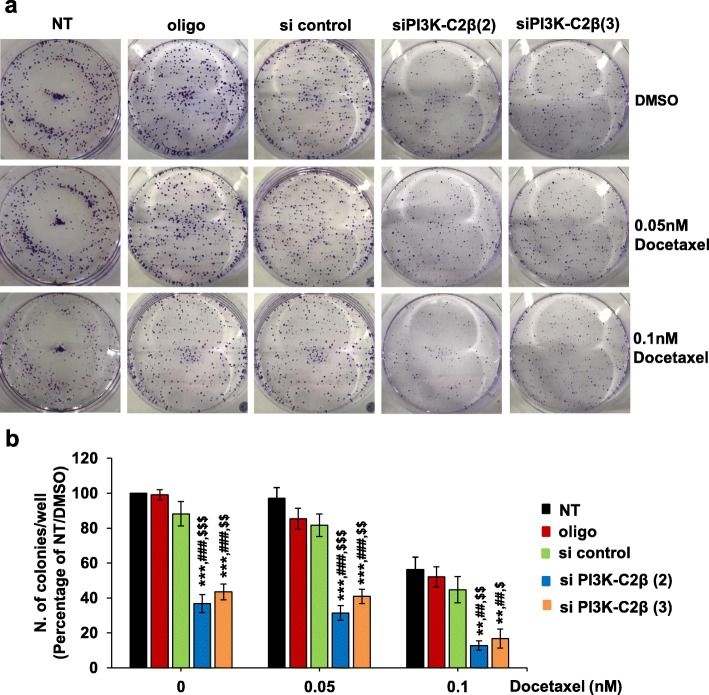
Combination of PI3K-C2β downregulation and docetaxel treatment strongly inhibits 2D colonies of HeLa cells in clonogenic assays. HeLa cells were transfected with siRNAs targeting PI3K-C2β, a control siRNA (si control) or treated with transfection reagent alone (oligo). Additional control cells were not transfected (NT). Cells were detached 24 h post transfection and plated as single cells (100 or 200 or 400 cells/well) in 6 well plates. Cells were incubated in complete media for 7 days in the presence of the indicated concentrations of docetaxel (or vehicle, DMSO) before being fixed and stained with crystal violet. Representative images of 2D colonies at the end of the experiment are shown in (**a**). Data in (**b**) indicate the number of colonies/well expressed as percentage of colonies from NT cells treated with DMSO and are means ± s.e.m. of *n* = 5 independent experiments performed in duplicate. **p < 0.01, ***p < 0.001 vs corresponding NT; ^##^*p* < 0.01, ^###^*p* < 0.001 vs corresponding oligo; ^$^*p* < 0.05, ^$$^*p* < 0.01, ^$$$^*p* < 0.001 vs corresponding si control

Finally, we assessed the effect of docetaxel treatment on the ability of PC3 and HeLa cells to form colonies in clonogenic assays. Consistent with our previous data (Fig. 1 and Additional file 3: Figure S2), both stable (Fig. 4a, b) and transient (Fig. 4c) downregulation of PI3K-C2β reduced the number of colonies in PC3 cells treated with vehicle. Treatment with increasing concentrations of docetaxel reduced the number of colonies in a dose-dependent manner in parental cells and all stable cell lines (Fig. 4b) as well as in transfected cells and their corresponding control cells (Fig. 4c). Combination of stable (Fig. 4a, b) or transient (Fig. 4c) PI3K-C2β downregulation with either 0.25 nM or 0.5 nM docetaxel strongly reduced the number of colonies, with almost complete inhibition of colonies formation in cells lacking the enzyme and treated with 0.5 nM docetaxel (Fig. 4a-c). Similar results were obtained upon PI3K-C2α downregulation (Additional file 9: Figure S8b). Consistent with data obtained in PC3 cells, downregulation of PI3K-C2β in HeLa cells also reduced the number of colonies and combination of transient PI3K-C2β downregulation with low docetaxel treatment almost completely blocked colonies formation (Fig. 5a, b and Additional file 10: Figure S9).

**Fig. 6:**
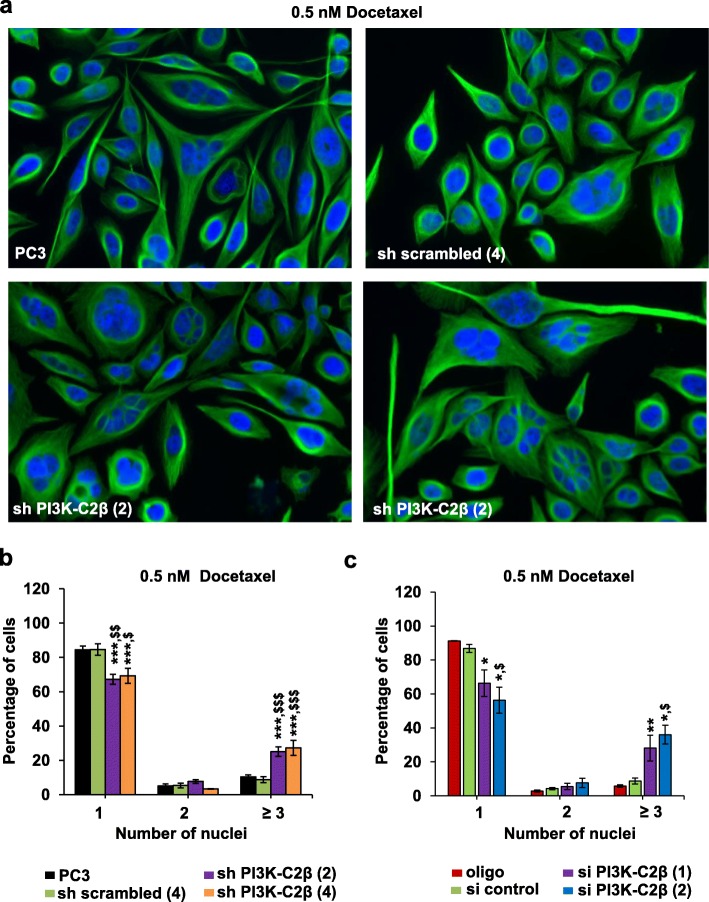
PI3K-C2β downregulation enhances docetaxel-induced multi-nucleation. (**a-c**) PC3 and the indicated stable cell lines were plated on coverslips (**a, b**). Alternatively, PC3 cells plated on coverslips were transfected with the indicated siRNAs or transfection reagent alone (**c**). The day after plating (**a, b**) or after transfection (**c**), cells were treated with 0.5 nM docetaxel (or corresponding amount of DMSO) and incubated for further 72 h. Cells were then fixed and stained with anti α-tubulin (green) and DAPI. Representative images of the indicated stable cells treated with 0.5 nM docetaxel are shown in (**a**). Graphs in (**b**, **c**) indicate the number of docetaxel-treated cells containing *n* = 1, n = 2 or n ≥ 3 (multi-nucleated) nuclei, expressed as percentage of total number of cells. Results from corresponding cells treated with vehicle alone are presented in Additional file 11: Figure S10a, b. Data in (**b**) are means ± s.e.m. of *n* = 6 (PC3 and sh scrambled), *n* = 4 [sh PI3K-C2β (3)] and *n* = 3 [sh PI3K-C2β (4)] independent experiments. The total numbers of cells analysed in these experiments were as follows: PC3 DMSO: 6254; PC3 docetaxel: 4250; sh scrambled (4) DMSO: 5942; sh scrambled (4) docetaxel: 3778; sh PI3K-C2β (3) DMSO: 3994; sh PI3K-C2β (3) docetaxel: 2773; sh PI3K-C2β (4) DMSO: 2372; sh PI3K-C2β (3) docetaxel: 2013. ***p < 0.001 vs corresponding PC3; ^$^*p* < 0.05, ^$$^*p* < 0.01, ^$$$^*p* < 0001 vs corresponding sh scrambled (4) (one tailed, unpaired t-Test with Welch’s correction). Data in (**c**) are means ± s.e.m. of *n* = 3–5 independent experiments. The total numbers of cells analysed in these experiments were as follows: oligo DMSO: 3553; oligo docetaxel: 2266; si control DMSO: 3986; si control docetaxel: 2746; si PI3K-C2β (1) DMSO: 3571; si PI3K-C2β (1) docetaxel: 2734; si PI3K-C2β (2) DMSO: 2239; si PI3K-C2β (2) docetaxel: 2136. *p < 0.05, **p < 0.01 vs corresponding oligo; ^$^*p* < 0.05 vs corresponding si control (one tailed, unpaired t-Test with Welch’s correction)

In an effort to define the mechanisms responsible for the enhanced effect of docetaxel in cells lacking PI3K-C2β, we determined the effect of downregulation of the enzyme on docetaxel-induced multi-nucleation. Both stable (Fig. 6a, b) and transient (Fig. 6c) downregulation of PI3K-C2β significantly increased the percentage of multi-nucleated PC3 cells and reduced the number of mono-nucleated cells upon docetaxel treatment. No difference in the percentage of mono-nucleated or bi-nucleated PC3 cells was observed in cells treated with DMSO whether they expressed or lacked PI3K-C2β stably (Additional file 11: Figure S10a) or transiently (Additional file 11: Figure S10b). No multi-nucleation was detected in PC3 cells transfected with si control or transfection reagent alone and treated with DMSO. Very few multi-nucleated cells were observed in PC3 cells upon transient PI3K-C2β downregulation in the absence of docetaxel although this was observed only in one experiment for cells transfected with si PI3K-C2β (1) and in two experiments for cells transfected with si PI3K-C2β (2), with percentages not reaching statistical significance [si PI3K-C2β (1): 0.09 ± 0.09; si PI3K-C2β (2): 0.17 ± 0.09]. Similarly, a significant increase in the percentage of multi-nucleated cells was detected in HeLa cells transfected with siRNAs targeting PI3K-C2β compared to control cells upon treatment with 0.25 nM docetaxel (Additional file 11: Figure S10c). No difference in multi-nucleation was detected between cells expressing or lacking the enzyme in the absence of docetaxel (Additional file 11: Figure S10c, DMSO).

Taken together these data indicate that combination of low concentrations of docetaxel and PI3K-C2β downregulation strongly reduce cancer cell growth and 2D colonies formation in vitro in a mechanism involving increased cellular multi-nucleation.

### Downregulation of PI3K-C2β potentiates the effect of docetaxel in vivo

**Fig. 7:**
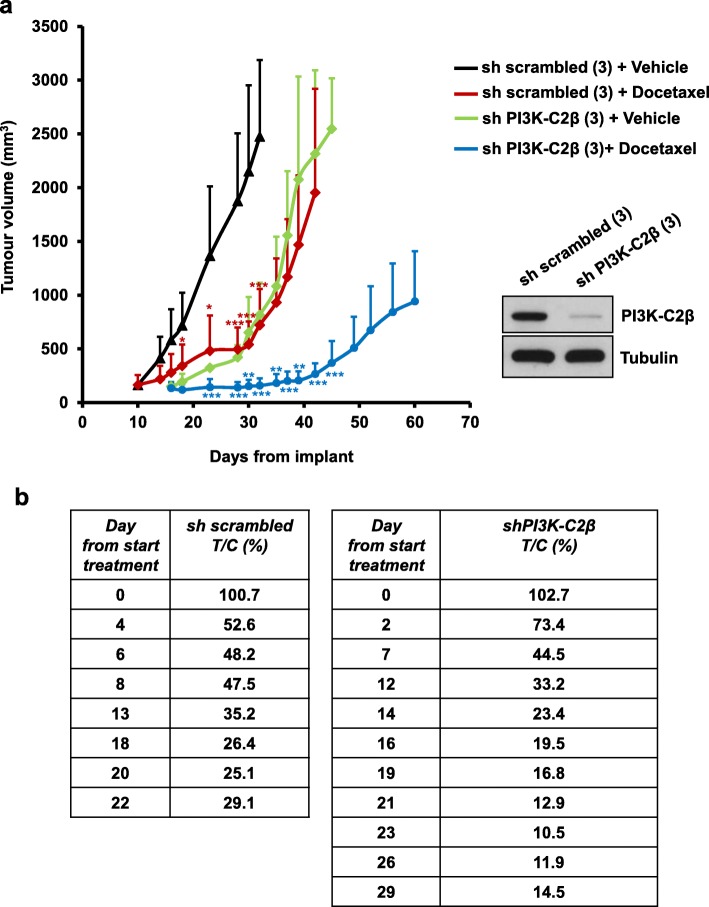
PI3K-C2β downregulation potentiates the effect of docetaxel in vivo. PC3 cells expressing [sh scrambled (3)] or lacking [sh PI3K-C2β (3)] PI3K-C2β were injected sub-cutaneously into the flanks of nude mice. Once tumours reached the pre-assigned size (150 mm^3^; sh scrambled: 10 days after implant, sh PI3K-C2β:16 days after implant), mice were treated with 3 mg/kg docetaxel or vehicle. **a** Tumour volumes were measured at the indicated times after implant. Data are from *n* = 7 mice per treatment. *p < 0.05, **p < 0.01, ***p < 0.001 vs corresponding vehicle. Blot indicating expression levels of PI3K-C2β in cells used for implant is also shown. Tubulin was used as loading control. **b** Data indicate T/C (%) values for mice bearing either sh scrambled or sh PI3K-C2β cells, measured at the indicated days from start of treatment (day 10 from implant for mice bearing sh scrambled cells; day 16 from implant for mice bearing sh PI3K-C2β cells). T/C (%) values were measured at the indicated days using the formula: weights of tumours from docetaxel-treated (T) mice/weights of tumours from vehicle-treated (C) X100

To investigate whether combination of PI3K-C2β downregulation and docetaxel treatment was also able to affect cancer cell growth in vivo, stable PC3 cells lacking PI3K-C2β (sh PI3K-C2β, clone 3) and corresponding control cells (sh scrambled, clone 3) were implanted in the flanks of nude mice. Once tumours reached the pre-assigned size (approximately 150 mm^3^), mice were treated with a low concentration of docetaxel (3 mg/kg) or vehicle control. Control, sh scrambled, cells generated tumours rapidly, with tumours reaching the pre-assigned size within 10 days from implant (Fig. 7a). Treatment of these mice with docetaxel efficiently delayed tumour growth and mice survived 10 days longer than corresponding mice treated with vehicle alone. When we analysed growth of tumours from cells lacking PI3K-C2β, first we observed that these cells required six additional days to form tumours of the pre-assigned size to start docetaxel treatment (Fig. 7a), indicating that downregulation of the enzyme delayed tumours growth in the first weeks after cells implant in vivo. Strikingly, when mice bearing sh PI3K-C2β cells were treated with docetaxel, tumours growth was delayed and strongly reduced. In fact, tumours lacking PI3K-C2β and treated with docetaxel barely increased in size in the first weeks of treatment and analysis of T/C (%) further indicated that tumour growth was strongly reduced in these mice (Fig. 7b). The enhanced activity of docetaxel in mice bearing shPI3K-C2β cells was not associated with increased toxicity as judged by macroscopic examination of animals during treatment and by constant monitoring of body weight, which did not change significantly in these mice compared to the corresponding vehicle-treated animals.

These data indicate that downregulation of PI3K-C2β potentiates the effect of docetaxel, resulting in strong inhibition of tumour growth in vivo.

## Discussion

### A novel role for PI3K-C2β in cancer cell mitosis

In this study, we identify a novel role for the class II PI3K isoform PI3K-C2β in regulation of cancer cell mitosis. Specifically, we report that downregulation of this enzyme delays cancer cell division, resulting in reduced ability of the cells to form 2D colonies in vitro and delayed tumour growth at least in the first weeks after cells implant in vivo.

Following initial studies from several laboratories, including our own, that first investigated the intracellular functions of the class II PI3K isoforms [30, 51, 53], our understanding of the physiological roles of these enzymes has improved massively in recent years, mostly because of the development of specific transgenic mouse models [62–70]. Knock-out animal models have established the central role of PI3K-C2α during embryogenesis, with ablation of *PIK3C2A* resulting in embryonic death due to defective vasculogenesis [63] and cilium formation [64]. Additional roles in platelets were also reported [66–68]. Characterisation of knock-out and knock-in PI3K-C2β mice, on the other hand, revealed that removal [62] or expression of a catalytic inactive form [69] of the enzyme did not affect viability. Enhanced insulin sensitivity of knock-in mice suggested a role for PI3K-C2β in insulin signalling regulation [69]. Finally, generation of PI3K-C2γ knock-out mice revealed its involvement in regulation of insulin signalling in hepatic cells [70]. So far, however, these models have provided little information on the potential involvement of class II PI3Ks in cancer development and/or progression. Crossing of heterozygous PI3K-C2α knock-out mice with transgenic models of breast cancer unveiled a complex role for this isoform, with reduction of PI3K-C2α levels resulting in initial delayed tumour growth followed by selection of fast growing cells and accelerated tumour growth [44]. While the impact of genetic ablation or inactivation of PI3K-C2β on transgenic cancer models has not been assessed yet, evidence now supports the conclusion that PI3K-C2β might play a role in several cancer types [46–55], mainly through regulation of cancer cell migration [46, 50–53], invasion [50, 52] and metastasis formation [50, 54]. Data on the potential involvement of this enzyme in cancer cell growth and proliferation are less clear, generally. Original data indicated reduced growth of small cell lung carcinoma H-69 cells expressing a dominant negative PI3K-C2β upon stimulation with stem cell factor but not with insulin or fibroblast growth factor-2 [71]. Downregulation of the enzyme also reduced proliferation in U937 cells [72] while its overexpression in A-431 cells increased proliferation [48]. On the other hand, downregulation of PI3K-C2β did not affect growth of adherent neuroblastoma cells but it reduced their anchorage-independent growth and tumour growth in vivo [55]. Similarly, we reported that PI3K-C2β downregulation did not affect growth of breast cancer cells in normal growing conditions while it reduced their growth upon stimulation with 17β-Oestradiol or heregulin B1 and in soft agar assays [50]. Furthermore, we observed that downregulation of PI3K-C2β reduced tumours growth in vivo when cells were injected into the mammary fat pad of nude mice but not when cells were injected subcutaneously [50]. Taken together, these data suggested that PI3K-C2β might be involved in cell growth/proliferation upon selective cellular stimulation or in specific cellular contexts.

Results from our study have unveiled a more complex contribution of PI3K-C2β to cancer cell growth. First, our observation that PI3K-C2β downregulation delayed progression from G2/M to G1 phase of the cell cycle following nocodazole block suggested a role for the enzyme during these phases of the cell cycle in PC3 cells. This would be consistent with previous data reporting activation of the enzyme during G2/M transition in HL-60 cells [45]. Time-lapse analyses confirmed a delay in mitosis progression upon downregulation of PI3K-C2β in both PC3 and HeLa cells. Specifically, time lapse analyses in PC3 cells suggested a potential contribution of the enzyme to distinct phases of mitosis, as both early and late stages of the process appeared to be delayed upon downregulation of the enzyme. Some of us previously reported that downregulation of the other class II PI3K, PI3K-C2α, delayed time required to progress from round up to anaphase and resulted in altered kinetochore-microtubule organisation, increased chromosome plate width and reduced metaphase spindle length [44]. These data revealed a role for PI3K-C2α during spindle assembly and anaphase onset during mitosis through a mechanism independent from its catalytic activity [44]. Consistent with this, this study reported that tumour onset was delayed in a transgenic mouse model of mammary carcinogenesis upon crossing with heterozygous *PIK3C2A*^+/−^ mice but this was then followed by a progressively increased rate of tumour formation in these mice [44]. Our observation that PI3K-C2α but not PI3K-C2β downregulation increased the percentage of apoptotic PC3 cells slightly, together with data indicating that downregulation of the two enzymes affected PC3 cell numbers with a different time course suggest independent and non-redundant roles for the two PI3Ks during mitosis. However, more detailed and specific studies are now required to determine the specific role of PI3K-C2β and its relative contribution to mitosis progression, in particular compared to PI3K-C2α. In this respect, our observation that PI3K-C2β downregulation in PC3 cells per se did not induce multi-nucleation and increased the time required for daughter cells to fully separate might suggest a potential role for this enzyme during late cytokinesis, for full abscission and separation of the two daughter cells. As hVps34-mediated synthesis of PtdIns3*P* at the midbody of dividing cells is critical to recruit proteins involved in the abscission process [43, 73] and PI3K-C2β is also known to regulate pools of this phosphoinositide [46], it would be tempting to speculate that PI3K-C2β might mediate the synthesis of an additional, localised pool of PtdIns3*P* at the midbody which contributes to full abscission. As our study was performed using selective siRNA-mediated downregulation of PI3K-C2β, however, it remains to be established whether the lipid activity of the enzyme is in fact required for its role during cell division.

We further show that the ability of cells lacking PI3K-C2β to form 2D colonies, when plated as single cells in clonogenic assays, is reduced strongly compared to control cells. Interestingly, it has been reported that the time required to complete abscission increases when cells density decreases because of changes in tension within the intercellular bridge [74]. These data suggest that the delayed cell division induced by PI3K-C2β downregulation might be exacerbated when single cells are plated and left to grow as colonies, resulting in the pronounced effect detected in clonogenic assays. It would be interesting to determine whether this also contributes to some of the differences previously detected between anchorage-dependent and anchorage-independent growth [50, 55].

Taken together, our study indicates that downregulation of PI3K-C2β reduces the ability of cancer cells to form colonies in clonogenic assays by delaying mitosis progression. Further studies are now required to establish the specific contribution of PI3K-C2β to this process and its specific mechanism of action and whether PI3K-C2β controls mitosis selectively in cancer cells.

### Downregulation of PI3K-C2β potentiates the effect of docetaxel in vitro and in vivo

Our study demonstrate that PI3K-C2β downregulation potentiates the effect of the microtubule stabilizing/anti-mitotic agent docetaxel on cancer cell growth in vitro and tumour growth in vivo. Specifically, we show that combination of PI3K-C2β downregulation with low concentrations of docetaxel strongly reduced 2D colonies formation in vitro and growth of prostate cancer cells in a xenograft model in vivo. Mechanistically, we observed that downregulation of the enzyme increases docetaxel-mediated multi-nucleation. These data suggest that PI3K-C2β downregulation might sensitise the cells to the effect of the drug by prolonging the time required to complete mitosis, therefore maintaining the cells for a longer time in a cell cycle phase when they are more sensitive to docetaxel treatment.

Our observation that blockade of PI3K-C2β can potentiate the effect of docetaxel in prostate cancer cells might have potential important clinical implications. A recent study reporting on the global burden of cancer worldwide using the GLOBOCAN 2018 described prostate cancer as the second cancer type for incidence in men globally, the most diagnosed cancer type in men in 105 countries and the fifth leading cause of cancer-related deaths in men globally [75]. While the 5-year survival rate of non metastatic prostate cancer is 98.9%, the rate for patients diagnosed with metastatic prostate cancer is less than 30%. Androgen deprivation therapy is the standard strategy for metastatic prostate cancer but, following initial response, almost all patients progress to castration-resistant prostate cancer (CRPC) which remains the main cause of mortality for prostate cancer patients [76, 77]. Taxanes still remain the only chemotherapeutic class to demonstrate survival benefits in advanced prostate cancer consistently [76], with docetaxel routinely used as first line treatment [78]. In 2004, results from two clinical trials demonstrating longer overall survival for patients with metastatic CRPC treated with docetaxel versus mitoxantrone made docetaxel the standard of care [79, 80]. In fact, docetaxel remained the only life-prolonging agent for metastatic CRPC until 2010 [81, 82]. Additional treatment options were later approved for these patients [83, 84], although trials are still ongoing to establish the optimal treatment sequence and timing [78, 82]. Unfortunately, although taxane chemotherapy significantly prolongs survival, these therapies still present several limitations, including development of serious side effects and mechanisms of resistance. Our observation of a potentiation of the effects of docetaxel in cells lacking PI3K-C2β suggests that simultaneous blockade of PI3K-C2β might represent a novel strategy to increase efficacy of the anti-mitotic drug, possibly resulting in fewer side effects. Whether simultaneous inhibition of PI3K-C2β might also prevent the development of mechanisms of resistance remains to be established. In this respect it is worth mentioning that a recent study reported that, while the majority of giant, multi-nucleated PC3 cells derived from docetaxel treatment eventually die by apoptosis, a small percentage of these cells can escape apoptosis and generate new mono-nucleated cells through neiosis [85], contributing to resistance development. Additional studies therefore will be required to understand fully the effect of simultaneous blockade of PI3K-C2β and docetaxel treatment in longer terms.

As selective inhibitors of PI3K-C2β are not commercially available, our data were obtained in cells upon downregulation of the enzyme. Whether the enzymatic activity of PI3K-C2β is required and therefore whether chemical inhibition of the enzyme would achieve the same effects in vitro and in vivo remains to be established. In this respect it is worth mentioning that, while selective class I PI3K inhibitors have been developed over the years and are being currently tested in clinical trials, with a selective p110δ approved for use in specific haematological cancers [86–88], development of selective inhibitors for the class II isoforms is lagging behind [89]. It is likely that the increasing evidence supporting the conclusion that PI3K-C2β regulates several cellular functions in distinct cancer types will raise more interest towards the development of much needed selective inhibitors of this enzyme.

## Conclusions

This study reported that the class II PI3K isoform PI3K-C2β is involved in regulation of mitotic progression. Downregulation of the enzyme delayed cancer cell division, resulting in reduced ability of the cells to form 2D colonies in clonogenic assays in vitro and in delayed growth of a prostate cancer xenograft model in vivo during the first weeks after cells implant. Moreover, our data indicated that PI3K-C2β downregulation in combination with low concentrations of docetaxel almost completely prevented 2D colonies formation in vitro and strongly inhibited tumour growth in vivo. These data suggest that blockade of PI3K-C2β might represent a novel strategy to improve efficacy of docetaxel.

## Supplementary information

**Additional file 1: Table S1.** Inhibition of p110β increases the percentage of cells in the G1 phase of cell cycle.

**Additional file 2: Figure S1.** Downregulation of PI3K-C2β in PC3 cells affects 2D colonies growth in clonogenic assays. The indicated stable clonal cell lines expressing (sh scrambled) or lacking (sh PI3K-C2β) PI3K-C2β were plated as single cells in 6 well plates and incubated in complete media. Representative images of 2D colonies at the indicated times after plating are shown.

**Additional file 3: Figure S2.** Transient downregulation of PI3K-C2β inhibits 2D colonies growth in clonogenic assays. PC3 cells were transfected with siRNAs targeting PI3K-C2β, siRNAs targeting PI3K-C2α or a non-targeting siRNA (si control). Additional control cells were non transfected (NT) or treated with transfection reagent alone (oligo). Cells were detached after 48 h, re-plated as single cells and incubated for further 10 days in complete media before being fixed and stained with crystal violet. Representative images of 2D colonies at the end of the experiments are shown.

**Additional file 4: Figure S3.** Downregulation of PI3K-C2β does not block proliferation and does not induce apoptosis. (**a, b**) The indicated cell lines were plated as single cells and grown as 2D colonies for 10 days. Fixed cells were analysed using IN Cell Analyzer 2200, as specified in the Methods section. Graph indicates the number of colonies, defined as groups of ≥50 cells (**a**), and number of cell aggregates containing <50 cells (**b**). Data are expressed as percentage of total number of cell colonies+aggregates (any groups of cells containing ≥2 cells). Data are means ± s.e.m. of *n* = 3–6 independent experiments. *p < 0.05 vs PC3; ^#^*p* < 0.05, ^##^*p* < 0.01 vs sh scrambled (4) (two tailed, unpaired t-Test with Welch’s correction). (**c**) The indicated cell lines were plated in complete media. The percentage of apoptotic cells was determined by Annexin V/FACS analysis after 24 h. Data are means ± s.e.m. of *n* = 3 (apart from sh scrambled, *n* = 2) independent experiments. (**d**) PC3 cells were transfected with the indicated siRNAs and then incubated for further 48 h before Annexin V/FACS analysis. Data indicate the percentage of apoptotic cells expressed as fold change of data from cells transfected with transfection reagent alone (oligo) and are means ± s.e.m. from *n* = 3 experiments. The average percentage of apoptotic “oligo” cells in these experiments was: 3.9 ± 0.5. *p < 0.05 vs oligo; ^#^*p* < 0.05, ^##^*p* < 0.01 vs si control (one tailed, unpaired t-Test with Welch’s correction).

**Additional file 5: Figure S4.** Downregulation of PI3K-C2β delays progression through G2/M phases following nocodazole treatment. The indicated stable cell lines were incubated in complete media supplemented with 100 nM nocodazole for 24 h. Cells that were still attached after treatment were transferred in complete media for further 2 h or 4 h. Data indicate the percentage of cells in each cell cycle phase at the indicated times after nocodazole treatment. Data are means ± s.e.m. of *n* = 3–4 independent experiments. *p < 0.05 (one-tailed unpaired t-Test with Welch’s correction).

**Additional file 6: Figure S5.** Downregulation of PI3K-C2β affects cell growth differently from other PI3K isoforms. (**a**, **b**) PC3 cells were transfected as indicated. In (**a**), cells were detached the day after transfection, re-plated and incubated for 120 h. In (**b**), the number of cells was assessed 72 h post-transfection. Data are expressed as percentage of si control-transfected cells and are from n = 3 [apart from NT, n = 2, si control, n = 4 and si PI3K-C2β(2), n = 4 in (**a**)] independent experiments. *p<0.05, **p<0.01 vs oligo; ^#^*p*<0.05 vs si control (**a**); **p<0.01 vs oligo, ^###^*p*<0.001 vs si control (**b**). In (**b**), p110β downregulation was confirmed by Western blotting, with GAPDH as loading control. (**c**) PC3 cells were treated with the p110β inhibitor GSK2636771, the pan-PI3K inhibitor LY294002 or vehicle (DMSO) for 72 h. Data are expressed as percentage of DMSO-treated cells and are from n = 3 independent experiments, except for 1µM GSK2636771 (n = 7). **p<0.01, ****p<0.0001 vs DMSO. (**d**) Number of PC3 cells transfected as indicated was assessed after 72 h and 96 h. Data are expressed as percentage of si control-transfected cells and are from n = 3-4 independent experiments. *p<0.05, **p<0.01 vs si control. (**e**) Transfected PC3 cells were treated with GSK2636771 (1µM) or DMSO after 24 h and for further 48 h. Data are expressed as percentage of si control-transfected cells treated with DMSO (si control/DMSO) and are from n = 3 independent experiments. *p<0.05; ^#^*p*<0.05, ^##^*p*<0.01 vs si control/DMSO. (**f**) HeLa cells were transfected as indicated and counted after 72 h. Data are expressed as percentage of si control-transfected cells and are from n = 4 independent experiments. **p<0.01, ***p<0.001 vs oligo; ^#^*p*<0.05, ^##^*p*<0.01 vs si control. All experiments were performed in duplicate. Data are from cell counting, are presented as means ± s.e.m. and were analysed by two tailed, unpaired t-Test with Welch’s correction [apart from data in (**e**), one-tailed].

**Additional file 7: Figure S6.** Low concentrations of docetaxel reduce cell numbers and induce multi-nucleation in PC3 cells. (**a**) PC3 cells were treated with the indicated concentrations of docetaxel for 72 h before cell counting. Control cells were treated with vehicle (DMSO) alone. Data are expressed as percentage of cells treated with DMSO and are means ± s.e.m. of *n* = 3–11 independent experiments performed in duplicate. **p < 0.01, ***p < 0.001, ****p < 0.0001 vs control (two tailed, unpaired t-Test with Welch’s correction). (**b**) PC3 cells were treated with the indicated concentrations of docetaxel or vehicle control (DMSO) for 48 h or 72 h before Annexin V/FACS analysis. Data indicate percentage of apoptotic cells expressed as fold change of results from cells treated with DMSO (control) and are means ± s.e.m. of *n* = 3 (48 h) and *n* = 6 (72 h) independent experiments performed in duplicate. The average percentage of apoptotic, DMSO-treated, cells in these experiments was: 7 ± 1 (48 h) and 7.4 ± 0.5 (72 h). *p < 0.05 vs corresponding control (one tailed, unpaired t-Test with Welch’s correction). (**c**) PC3 cells plated on coverslips were treated with 0.5 nM docetaxel (or corresponding amount of DMSO) and incubated for 72 h before being fixed and stained with anti α-tubulin (green) and DAPI. Representative images at different magnifications are shown. Arrows indicate multi-nucleated cells.

**Additional file 8: Figure S7.** Low concentrations of docetaxel induce multi-nucleation in HeLa cells. (**a**, **b**) HeLa cells were seeded onto coverslips and treated with the indicated concentrations of docetaxel or vehicle control (DMSO) for 72 h before being fixed and stained with anti α-tubulin (green) and DAPI. Representative images at different magnifications are shown in (**a**). Arrows indicate multi-nucleated cells. Graph in (**b**) indicate the number of cells containing *n* = 1, *n* = 2 or *n* ≥ 3 nuclei, expressed as percentage of total number of cells and are means ± s.e.m. of n = 3 (apart from 0.25 nM docetaxel, *n* = 2) independent experiments. The total numbers of cells analysed in these experiments were as follows: DMSO: 1800; 0.25 nM docetaxel: 936; 0.5 nM docetaxel: 746. *p < 0.05 vs corresponding DMSO. (**c**) HeLa cells were transfected with a control siRNA (si control) or treated with transfection reagent alone (oligo). Cells were detached 24 h post transfection and plated on coverslips in 12 well plates. The following day, cells were treated with the indicated concentrations of docetaxel or vehicle alone (DMSO) and incubated for further 48 h before fixing and staining. Data indicate the number of cells containing n = 1, *n* = 2 or *n* ≥ 3 nuclei, expressed as percentage of total number of cells and are means ± s.e.m. of *n* = 3 independent experiments. The total numbers of cells analysed in these experiments were as follows: oligo DMSO: 1532; oligo 0.25 nM docetaxel: 1291; oligo 0.5 nM docetaxel: 809; si control DMSO: 1171; si control 0.25 nM docetaxel: 1170; si control 0.5 nM docetaxel: 719. *p < 0.05, **p < 0.01, ***p < 0.001 vs corresponding DMSO.

**Additional file 9: Figure S8.** Combination of PI3K-C2α downregulation and docetaxel inhibits 2D colonies in clonogenic assays. (**a**) PC3 cells were transfected with a siRNA targeting PI3K-C2α or a non targeting siRNA. After 24 h, cells were incubated in complete media supplemented with 0.5 nM docetaxel or DMSO for further 48 h. The number of cells was assessed by cell counting. Data are expressed as percentage of cells transfected with si control and treated with DMSO and are means ± s.e.m. of n = 3 independent experiments performed in duplicate. **p < 0.01 vs si control/DMSO; ^##^*p* < 0.01 vs si PI3K-C2α/DMSO; ^$$^*p* < 0.01 vs si control/docetaxel (two tailed, unpaired t-Test with Welch’s correction). (**b**) PC3 were transfected with the indicated siRNAs or transfection reagent alone (oligo) for 24 h before being detached and plated as single cells. Cells were incubated in complete media for 10 days in the presence of the indicated concentrations of docetaxel (or vehicle) before being fixed and stained with crystal violet. Data indicate the number of colonies (> 65 cells) and are means ± s.e.m. of n = 3 independent experiments performed in duplicate. *p < 0.05, **p < 0.01 vs corresponding oligo; ^#^*p* < 0.05, ^##^*p* < 0.01 vs corresponding si control (two tailed, unpaired t-Test with Welch’s correction).

**Additional file 10: Figure S9.** Effect of combination of PI3K-C2β downregulation and docetaxel on 2D colonies of HeLa cells in clonogenic assays. HeLa cells were transfected with siRNAs targeting PI3K-C2β, a control siRNA (si control) or treated with transfection reagent alone (oligo). Not transfected (NT) cells were also used as additional control. Cells were detached 24 h post transfection and plated as single cells (100 or 200 or 400 cells/well) in 6 well plates in duplicate. Cells were incubated in complete media for 7 days in the absence (“NT”) or presence of the indicated concentrations of docetaxel (or vehicle, DMSO) before being fixed and stained with crystal violet. Representative images of 6 well plates are shown.

**Additional file 11: Figure S10.** Effect of PI3K-C2β downregulation and docetaxel on multi-nucleation. (**a**, **b**) The indicated cells were treated as described in Fig. 6. Graphs indicate the number of cells treated with DMSO (in parallel to cells treated with docetaxel, presented in Fig. 6) and containing n = 1 and n = 2 nuclei. Data are expressed as percentage of total number of cells and are means ± s.e.m. of n = 6 (PC3 and sh scrambled), n = 4 [sh PI3K-C2β (3)] and n = 3 [sh PI3K-C2β (4)] (**a**) or n = 4 (**b**) independent experiments. In these conditions, no difference in the percentage of multi-nucleated cells was detected between the cell lines [PC3: 0.2±0.08; sh scrambled: 0.07±0.05; sh PI3K-C2β (3): 0.26±0.2; sh PI3K-C2β (4): 0.04±0.04] and no multi-nucleated cells were detected in cells transfected with transfection reagent or with si control. Multi-nucleation was detected only in one out of three experiments for si PI3K-C2β (1)-transfected cells [average percentage: 0.5±0.5, n = 3] and in two out of three experiments for cells transfected with si PI3K-C2β (2) [average percentage: 0.4±0.3, n = 3]. (**c**) HeLa cells were transfected as indicated, detached 24 h post transfection and plated on coverslips. The following day, cells were treated with 0.25nM docetaxel or DMSO and incubated for further 72 h before fixing and staining. Data indicate the number of cells containing n = 1, n = 2 or n≥3 nuclei, expressed as percentage of total number of cells and are means ± s.e.m. of n = 5 independent experiments [apart from si PI3K-C2β (3) DMSO, n = 4]. The total numbers of cells analysed in these experiments were as follows: oligo DMSO: 2582; oligo 0.25nM docetaxel: 1658; si control DMSO: 1658; si control 0.25nM docetaxel: 1122; si PI3K-C2β (2) DMSO: 1290; si PI3K-C2β (2) 0.25nM docetaxel: 711; si PI3K-C2β (3) DMSO: 846; si PI3K-C2β (3) 0.25nM docetaxel: 884. *p<0.05, **p<0.01, vs corresponding oligo; ^#^ *p*<0.05, ^###^*p*<0.001 vs corresponding si control.

## Data Availability

All data generated or analysed during this study are included in this manuscript and its supplementary information files.

## Abbreviations

CDK: Cyclin-dependent kinase
DAPI: 4′,6-diamidino-2-phenylindole
PBS-T: Phosphate buffer saline supplemented with 0.05% Tween-20
PFA: Paraformaldehyde
PI3K: Phosphoinositide 3-kinase
PtdIns3*P*: Phosphatidylinositol 3-phosphate
