# Supplementary material for: Downregulation of class II phosphoinositide 3-kinase PI3K-C2β delays cell division and potentiates the effect of docetaxel on cancer cell growth
Source: J Exp Clin Cancer Res. 2019 Nov 21;38:472. doi: 10.1186/s13046-019-1472-9 (PMC6873561; doi:10.1186/s13046-019-1472-9)
Supplement: Supplementary file 1 — Additional file 1: Table S1. Inhibition of p110β increases the percentage of cells in the G1 phase of cell cycle. [file 13046_2019_1472_MOESM1_ESM.docx]

**Supplementary Table 1. Inhibition of p110β increases the percentage of cells in the G1 phase of cell cycle**

|  | **subG1** | **G1** | **S** | **G2/M** |
| --- | --- | --- | --- | --- |
| **DMSO** | 0.5 ± 0.2 | 53.55 ± 1.62 | 21.9 ± 2.52 | 20.52 ± 0.60 |
| **GSK2636771** | 0.47 ± 0.17 | 64.47 ± 2.11** | 16.5 ± 1.98 | 16.52 ± 0.37** |

PC3 cells were treated with the selective p110β inhibitor (1µM) or vehicle for 72h. The percentage of cells in the indicated cell cycle phases were measured by PI staining/FACS analysis. Data are means ± s.e.m. from n=4 independent experiments. **p<0.01 vs corresponding DMSO.
