# Supplementary figures and images for: Downregulation of class II phosphoinositide 3-kinase PI3K-C2β delays cell division and potentiates the effect of docetaxel on cancer cell growth
Source: J Exp Clin Cancer Res. 2019 Nov 21;38:472. doi: 10.1186/s13046-019-1472-9 (PMC6873561; doi:10.1186/s13046-019-1472-9)

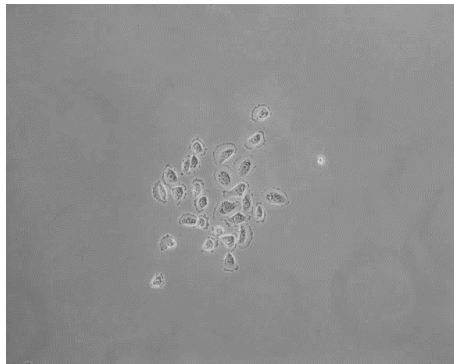

**sh scrambled (3)**

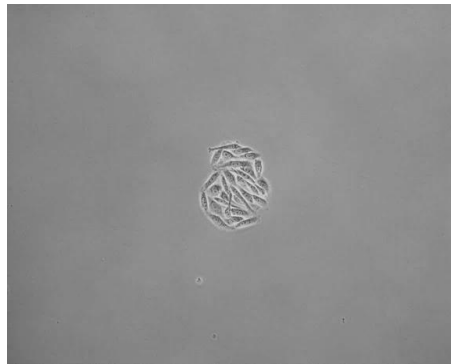

**sh PI3K-C2 $\beta$  (2)**

***Day 5***

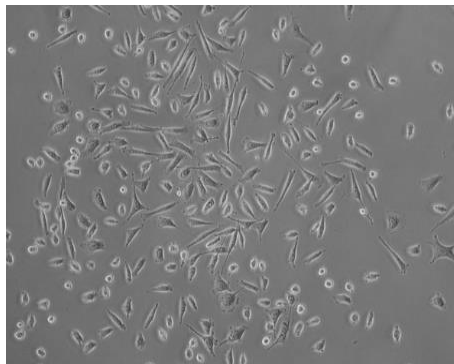

**sh scrambled (3)**

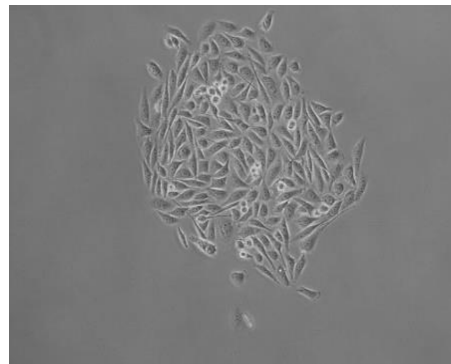

**sh PI3K-C2 $\beta$  (2)**

***Day 8***

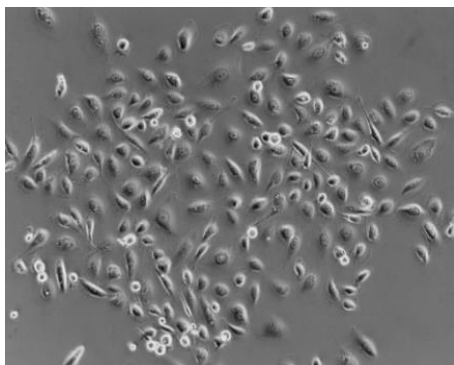

**sh scrambled (3)**

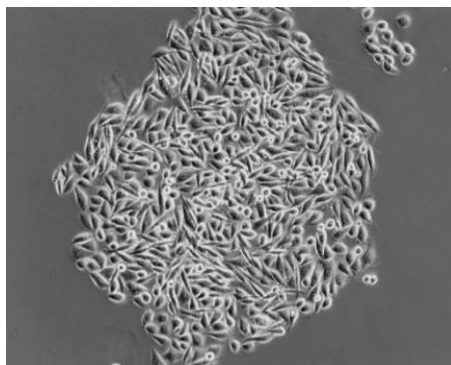

**sh PI3K-C2 $\beta$  (3)**

***Day 10***

Supplement: Supplementary file 2 — Additional file 2: Figure S1. Downregulation of PI3K-C2β in PC3 cells affects 2D colonies growth in clonogenic assays. The indicated stable clonal cell lines expressing (sh scrambled) or lacking (sh PI3K-C2β) PI3K-C2β were plated as single cells in 6 well plates and incubated in complete media. Representative images of 2D colonies at the indicated times after plating are shown. [file 13046_2019_1472_MOESM2_ESM.pdf]

**NT**

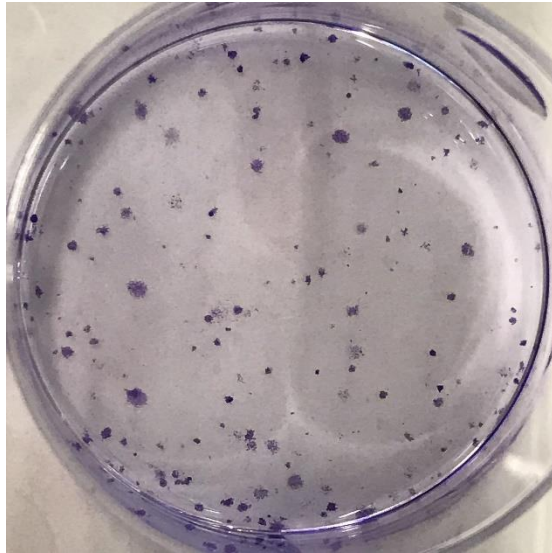

**oligo**

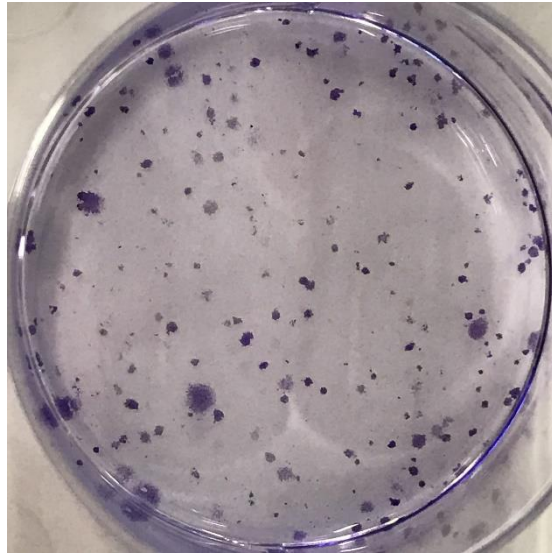

**si control**

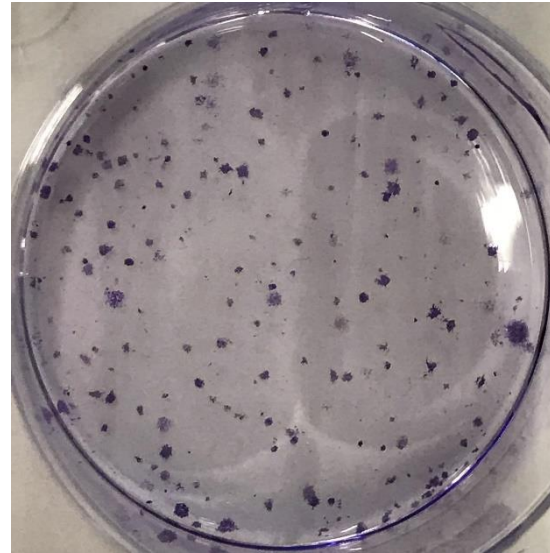

**si PI3K-C2 $\beta$  (1)**

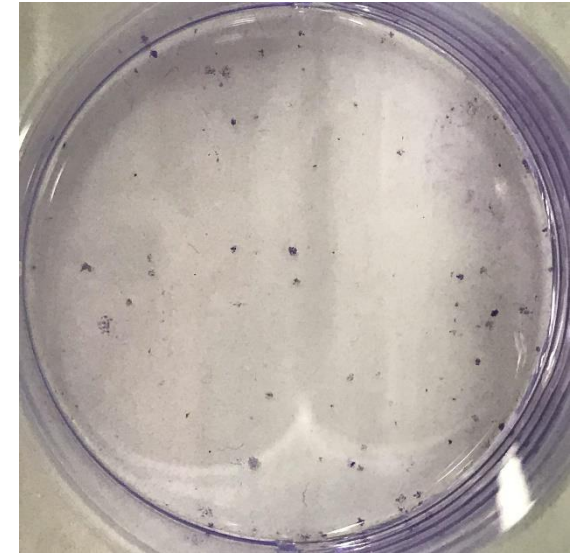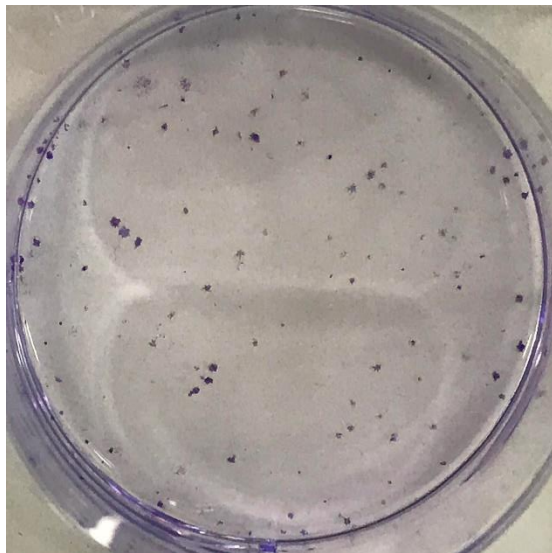

**si PI3K-C2 $\alpha$  (1)**

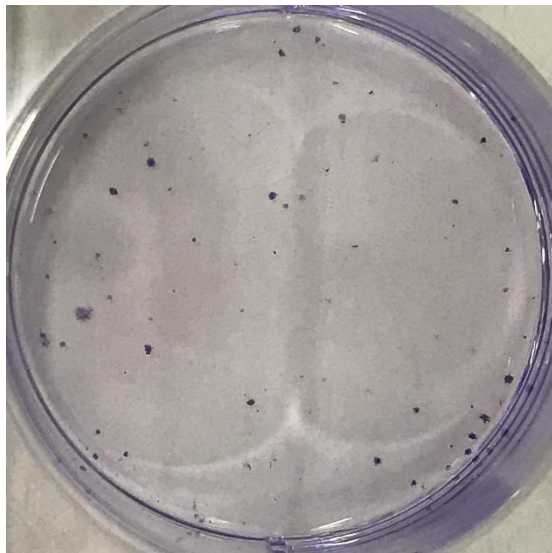

**si PI3K-C2 $\alpha$  (2)**

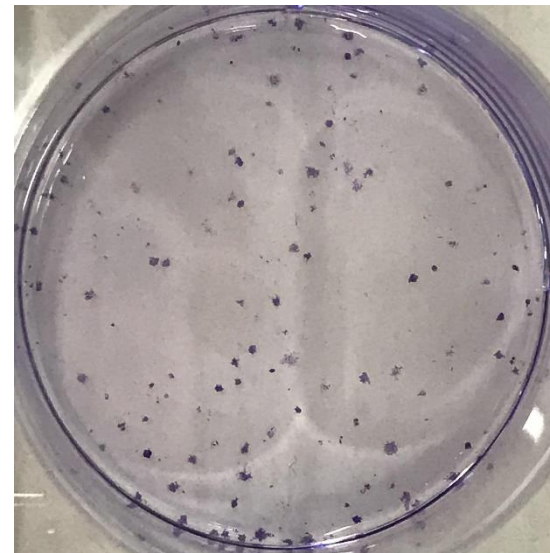

**si PI3K-C2 $\alpha$  (3)**

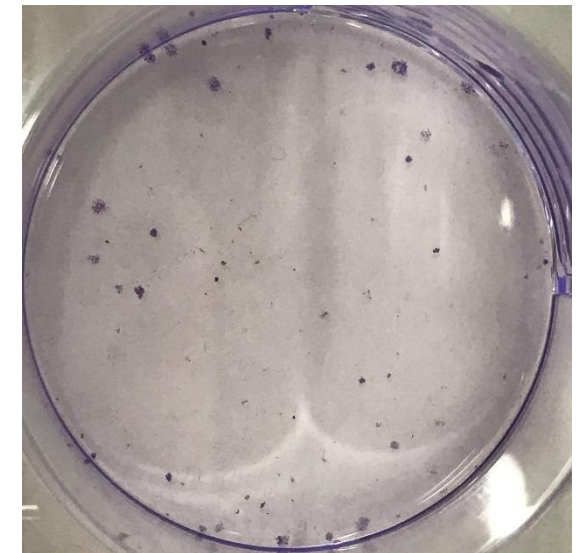

**si PI3K-C2 $\beta$  (2)**

Supplement: Supplementary file 3 — Additional file 3: Figure S2. Transient downregulation of PI3K-C2β inhibits 2D colonies growth in clonogenic assays. PC3 cells were transfected with siRNAs targeting PI3K-C2β, siRNAs targeting PI3K-C2α or a non-targeting siRNA (si control). Additional control cells were non transfected (NT) or treated with transfection reagent alone (oligo). Cells were detached after 48 h, re-plated as single cells and incubated for further 10 days in complete media before being fixed and stained with crystal violet. Representative images of 2D colonies at the end of the experiments are shown. [file 13046_2019_1472_MOESM3_ESM.pdf]

**a**

$\geq 50$  cells

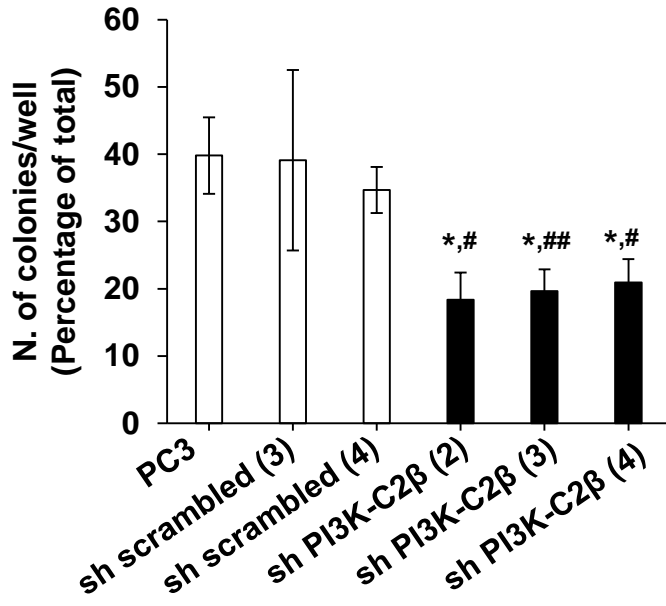

**b**

<50 cells

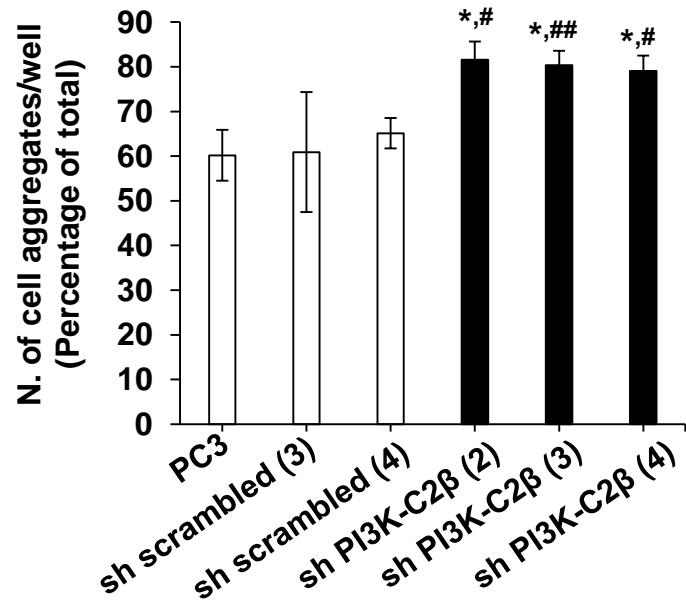

**c**

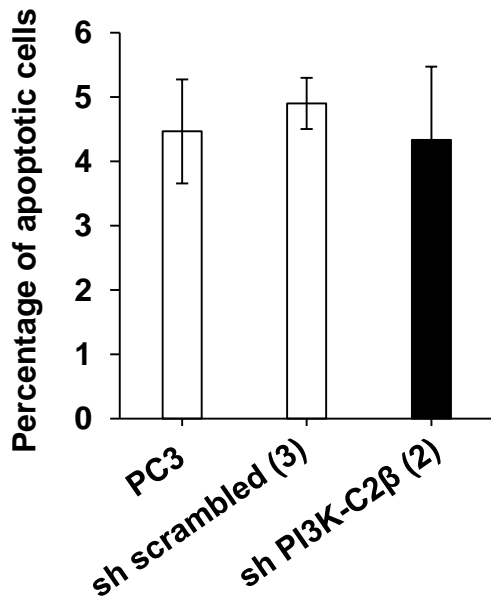

**d**

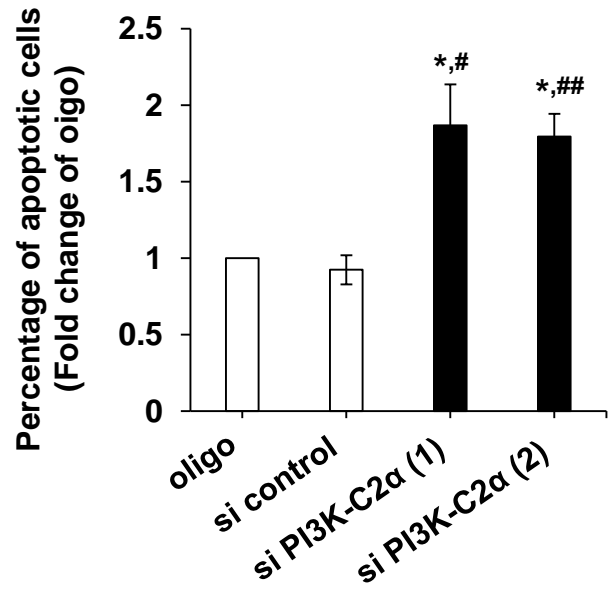

Supplement: Supplementary file 4 — Additional file 4: Figure S3. Downregulation of PI3K-C2β does not block proliferation and does not induce apoptosis. (a, b) The indicated cell lines were plated as single cells and grown as 2D colonies for 10 days. Fixed cells were analysed using IN Cell Analyzer 2200, as specified in the Methods section. Graph indicates the number of colonies, defined as groups of ≥50 cells (a), and number of cell aggregates containing <50 cells (b). Data are expressed as percentage of total number of cell colonies+aggregates (any groups of cells containing ≥2 cells). Data are means ± s.e.m. of n = 3–6 independent experiments. *p < 0.05 vs PC3; #p < 0.05, ##p < 0.01 vs sh scrambled (4) (two tailed, unpaired t-Test with Welch’s correction). (c) The indicated cell lines were plated in complete media. The percentage of apoptotic cells was determined by Annexin V/FACS analysis after 24 h. Data are means ± s.e.m. of n = 3 (apart from sh scrambled, n = 2) independent experiments. (d) PC3 cells were transfected with the indicated siRNAs and then incubated for further 48 h before Annexin V/FACS analysis. Data indicate the percentage of apoptotic cells expressed as fold change of data from cells transfected with transfection reagent alone (oligo) and are means ± s.e.m. from n = 3 experiments. The average percentage of apoptotic “oligo” cells in these experiments was: 3.9 ± 0.5. *p < 0.05 vs oligo; #p < 0.05, ##p < 0.01 vs si control (one tailed, unpaired t-Test with Welch’s correction). [file 13046_2019_1472_MOESM4_ESM.pdf]

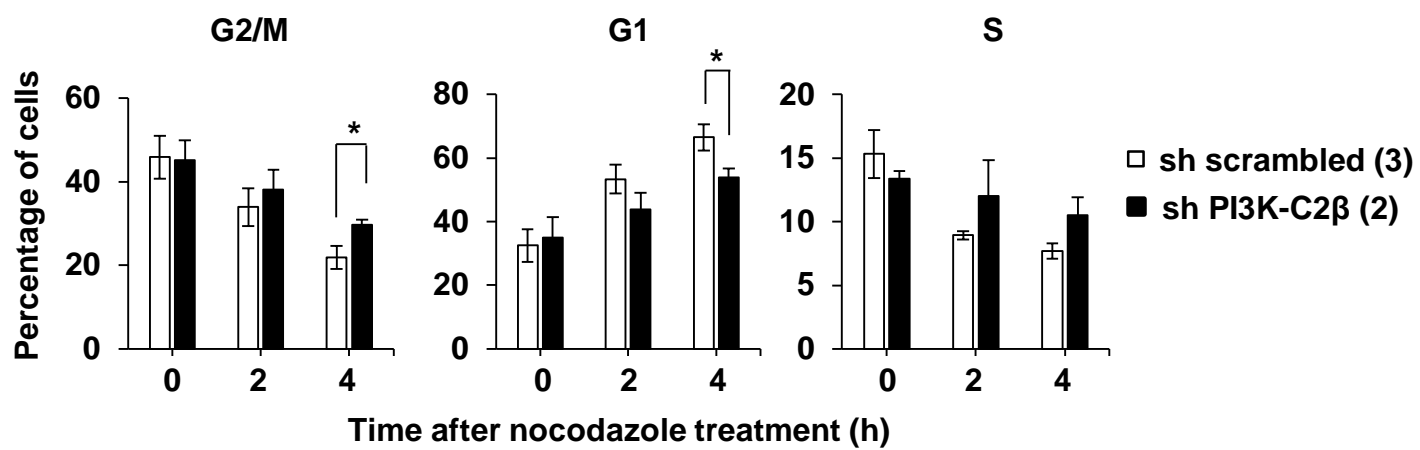

Supplement: Supplementary file 5 — Additional file 5: Figure S4. Downregulation of PI3K-C2β delays progression through G2/M phases following nocodazole treatment. The indicated stable cell lines were incubated in complete media supplemented with 100 nM nocodazole for 24 h. Cells that were still attached after treatment were transferred in complete media for further 2 h or 4 h. Data indicate the percentage of cells in each cell cycle phase at the indicated times after nocodazole treatment. Data are means ± s.e.m. of n = 3–4 independent experiments. *p < 0.05 (one-tailed unpaired t-Test with Welch’s correction). [file 13046_2019_1472_MOESM5_ESM.pdf]

**a**

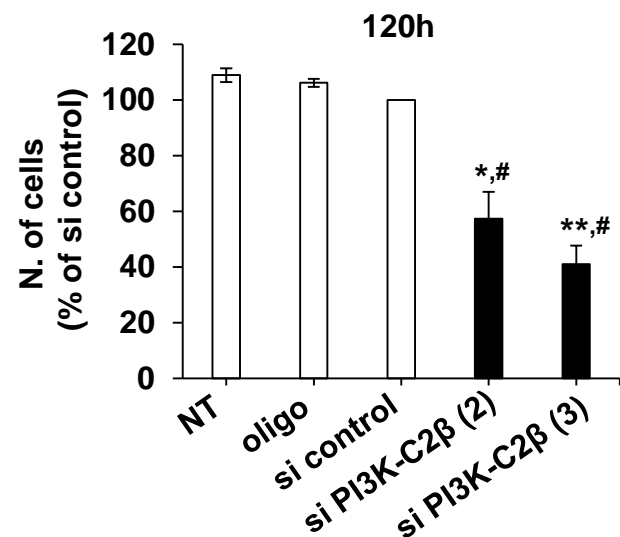

**b**

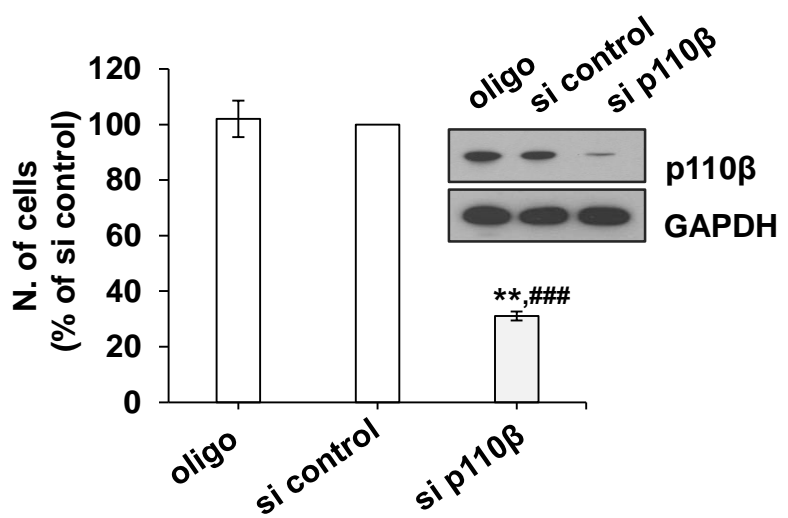

**c**

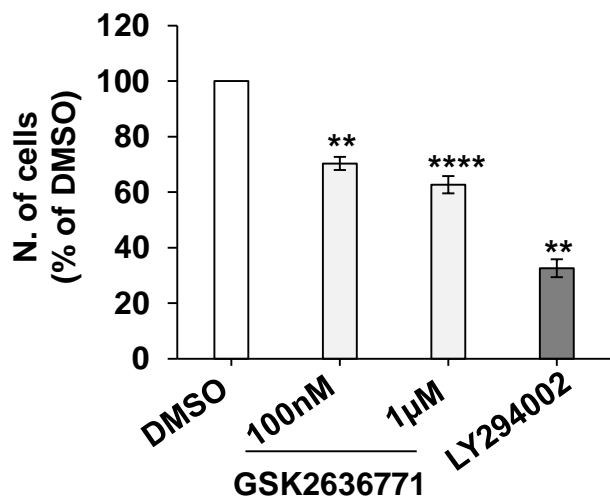

**d**

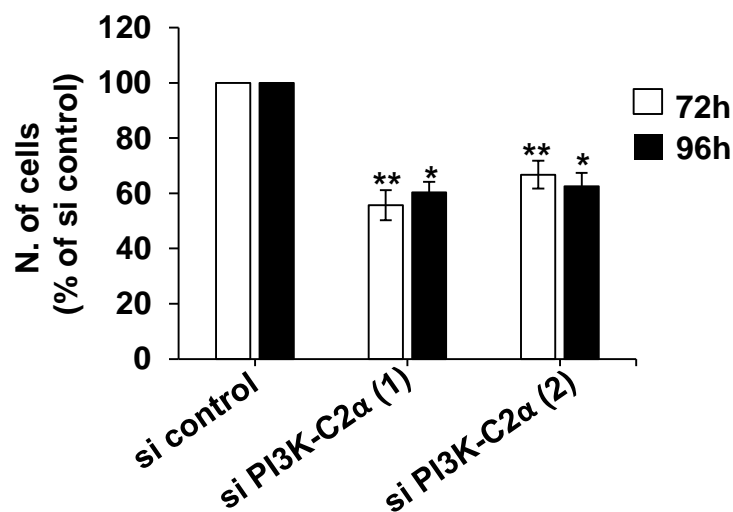

**e**

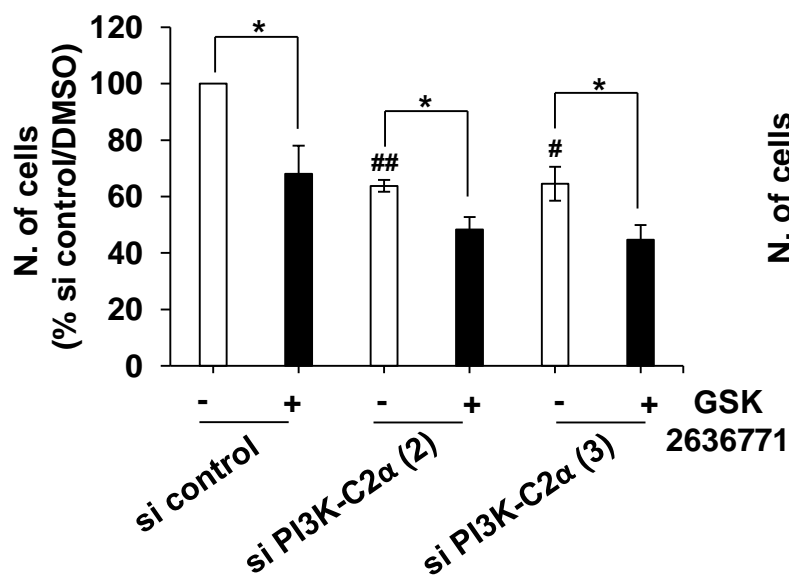

**f**

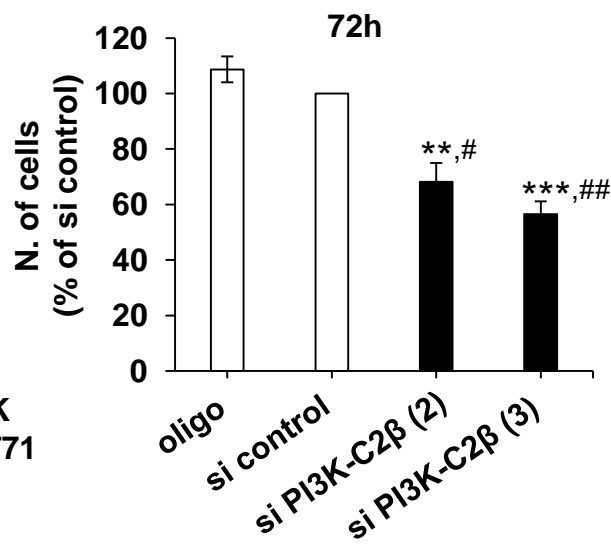

Supplement: Supplementary file 6 — Additional file 6: Figure S5. Downregulation of PI3K-C2β affects cell growth differently from other PI3K isoforms. (a, b) PC3 cells were transfected as indicated. In (a), cells were detached the day after transfection, re-plated and incubated for 120 h. In (b), the number of cells was assessed 72 h post-transfection. Data are expressed as percentage of si control-transfected cells and are from n = 3 [apart from NT, n = 2, si control, n = 4 and si PI3K-C2β(2), n = 4 in (a)] independent experiments. *p<0.05, **p<0.01 vs oligo; #p<0.05 vs si control (a); **p<0.01 vs oligo, ###p<0.001 vs si control (b). In (b), p110β downregulation was confirmed by Western blotting, with GAPDH as loading control. (c) PC3 cells were treated with the p110β inhibitor GSK2636771, the pan-PI3K inhibitor LY294002 or vehicle (DMSO) for 72 h. Data are expressed as percentage of DMSO-treated cells and are from n = 3 independent experiments, except for 1µM GSK2636771 (n = 7). **p<0.01, ****p<0.0001 vs DMSO. (d) Number of PC3 cells transfected as indicated was assessed after 72 h and 96 h. Data are expressed as percentage of si control-transfected cells and are from n = 3-4 independent experiments. *p<0.05, **p<0.01 vs si control. (e) Transfected PC3 cells were treated with GSK2636771 (1µM) or DMSO after 24 h and for further 48 h. Data are expressed as percentage of si control-transfected cells treated with DMSO (si control/DMSO) and are from n = 3 independent experiments. *p<0.05; #p<0.05, ##p<0.01 vs si control/DMSO. (f) HeLa cells were transfected as indicated and counted after 72 h. Data are expressed as percentage of si control-transfected cells and are from n = 4 independent experiments. **p<0.01, ***p<0.001 vs oligo; #p<0.05, ##p<0.01 vs si control. All experiments were performed in duplicate. Data are from cell counting, are presented as means ± s.e.m. and were analysed by two tailed, unpaired t-Test with Welch’s correction [apart from data in (e), one-tailed]. [file 13046_2019_1472_MOESM6_ESM.pdf]

**a**

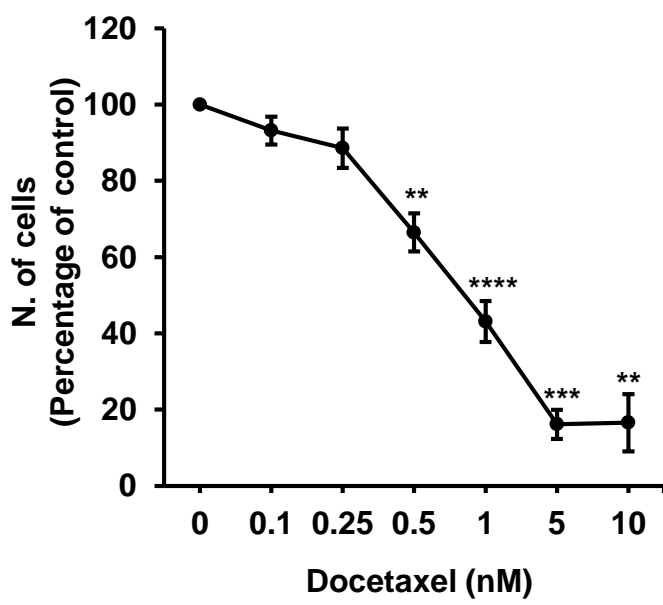

**b**

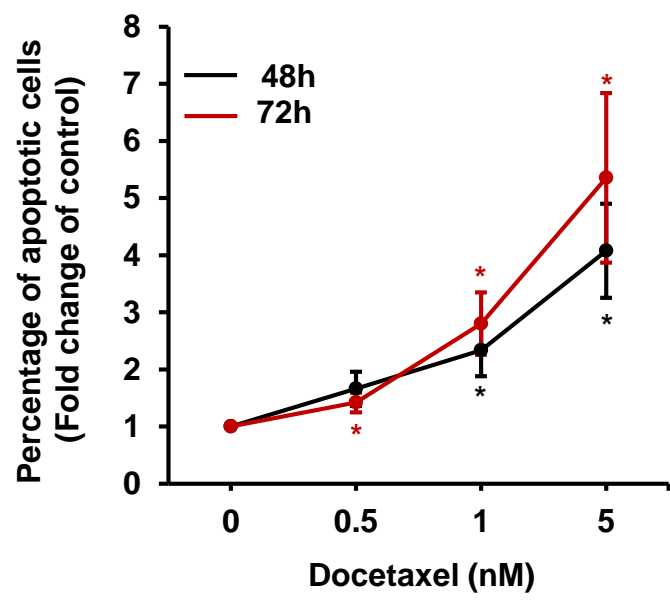

**c**

DMSO

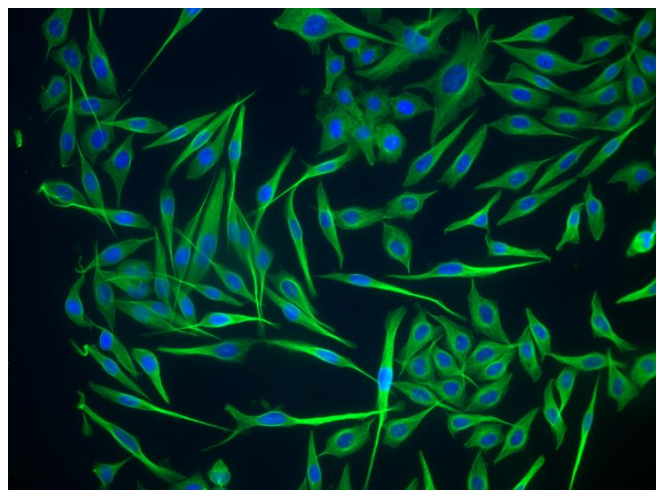

0.5nM Docetaxel

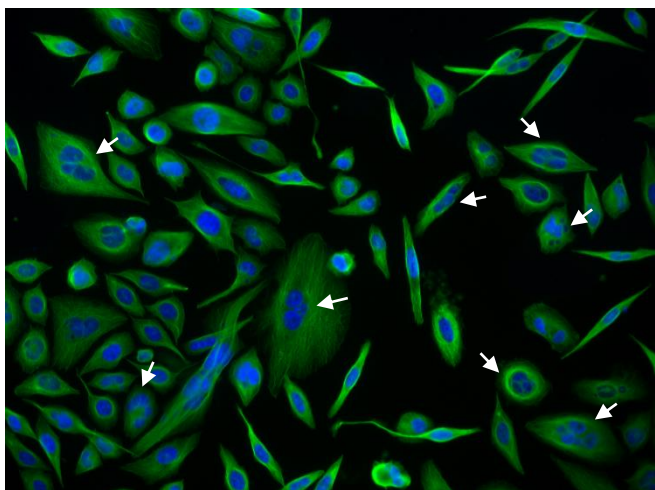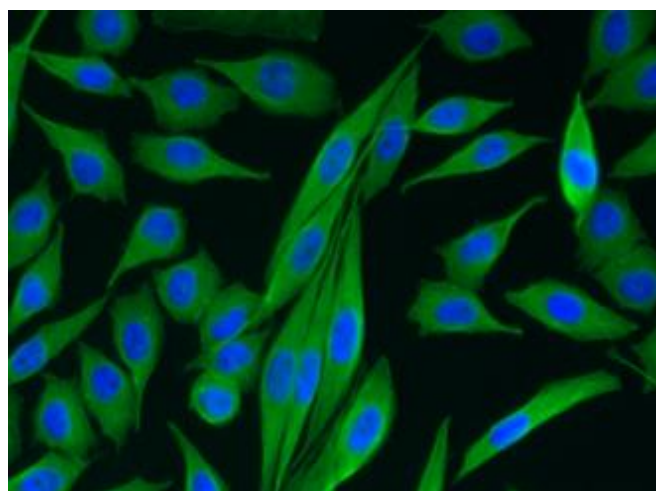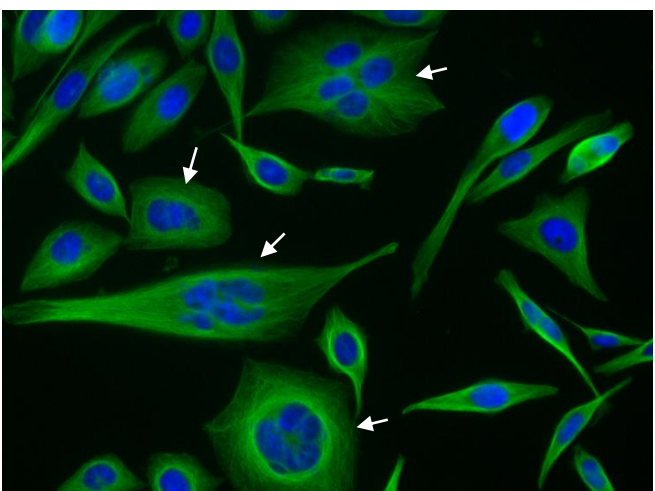

Supplement: Supplementary file 7 — Additional file 7: Figure S6. Low concentrations of docetaxel reduce cell numbers and induce multi-nucleation in PC3 cells. (a) PC3 cells were treated with the indicated concentrations of docetaxel for 72 h before cell counting. Control cells were treated with vehicle (DMSO) alone. Data are expressed as percentage of cells treated with DMSO and are means ± s.e.m. of n = 3–11 independent experiments performed in duplicate. **p < 0.01, ***p < 0.001, ****p < 0.0001 vs control (two tailed, unpaired t-Test with Welch’s correction). (b) PC3 cells were treated with the indicated concentrations of docetaxel or vehicle control (DMSO) for 48 h or 72 h before Annexin V/FACS analysis. Data indicate percentage of apoptotic cells expressed as fold change of results from cells treated with DMSO (control) and are means ± s.e.m. of n = 3 (48 h) and n = 6 (72 h) independent experiments performed in duplicate. The average percentage of apoptotic, DMSO-treated, cells in these experiments was: 7 ± 1 (48 h) and 7.4 ± 0.5 (72 h). *p < 0.05 vs corresponding control (one tailed, unpaired t-Test with Welch’s correction). (c) PC3 cells plated on coverslips were treated with 0.5 nM docetaxel (or corresponding amount of DMSO) and incubated for 72 h before being fixed and stained with anti α-tubulin (green) and DAPI. Representative images at different magnifications are shown. Arrows indicate multi-nucleated cells. [file 13046_2019_1472_MOESM7_ESM.pdf]

a

DMSO

0.5nM Docetaxel

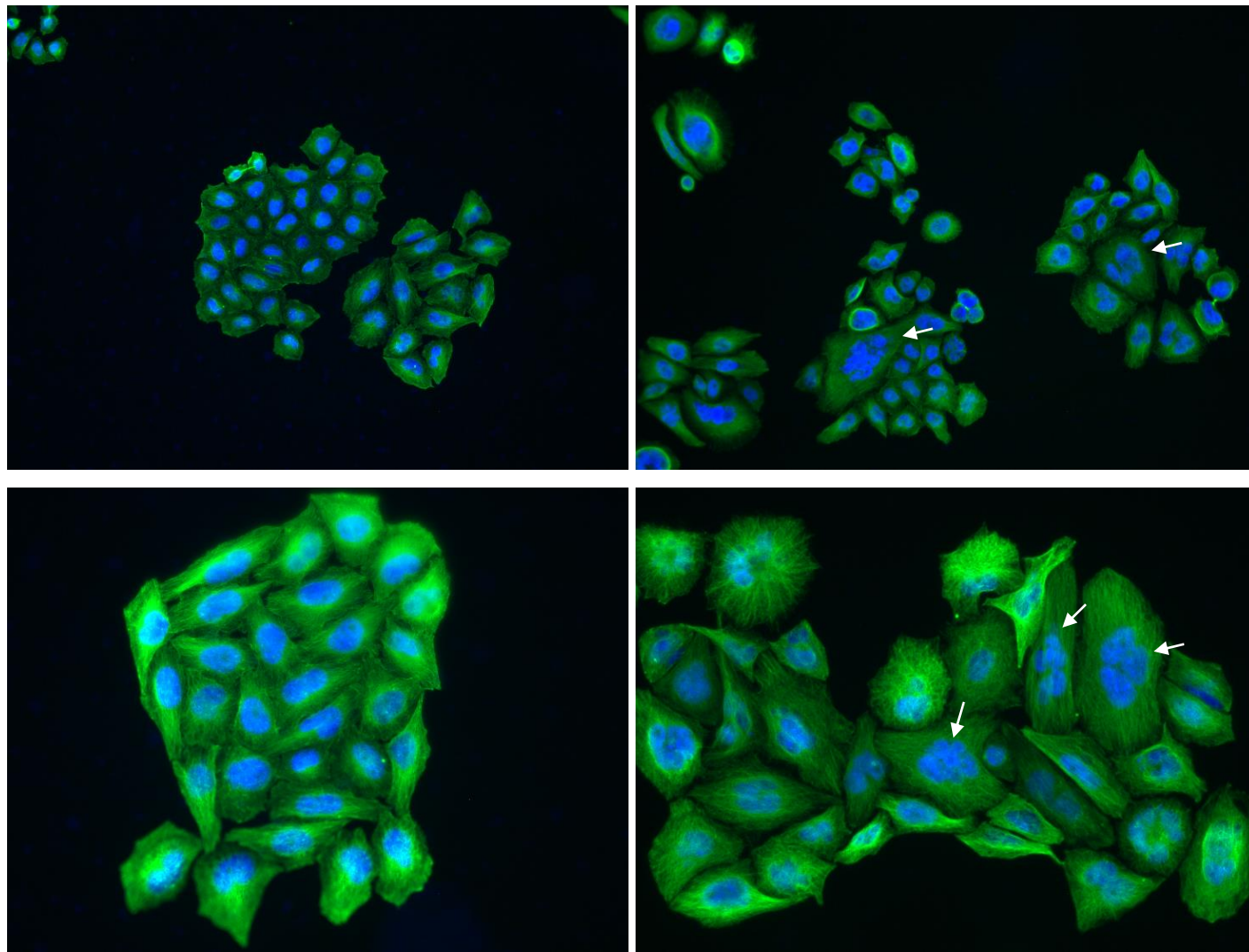

b

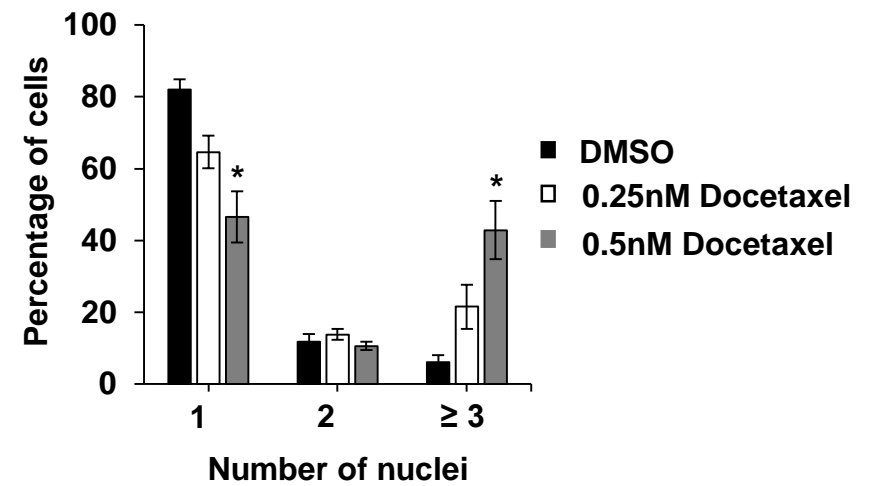

c

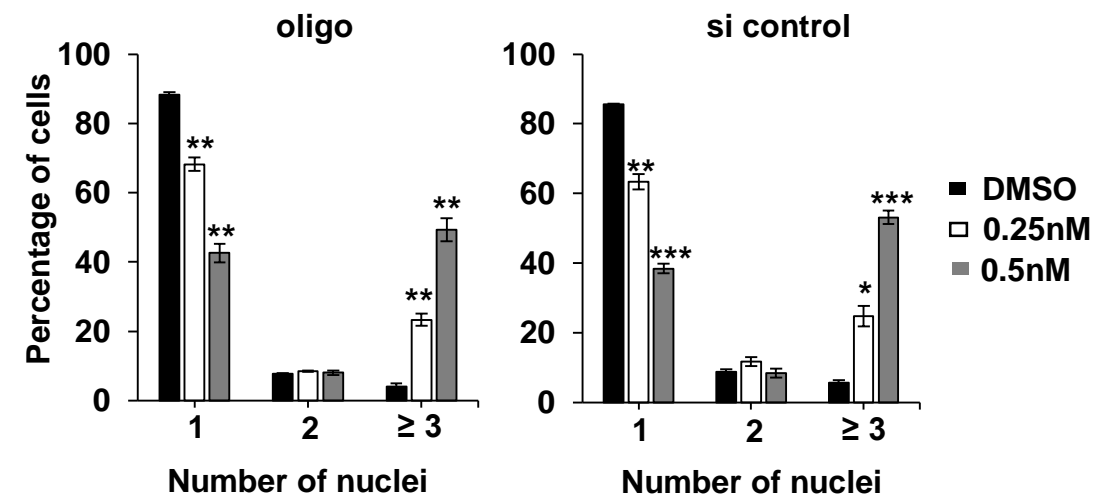

Supplement: Supplementary file 8 — Additional file 8: Figure S7. Low concentrations of docetaxel induce multi-nucleation in HeLa cells. (a, b) HeLa cells were seeded onto coverslips and treated with the indicated concentrations of docetaxel or vehicle control (DMSO) for 72 h before being fixed and stained with anti α-tubulin (green) and DAPI. Representative images at different magnifications are shown in (a). Arrows indicate multi-nucleated cells. Graph in (b) indicate the number of cells containing n = 1, n = 2 or n ≥ 3 nuclei, expressed as percentage of total number of cells and are means ± s.e.m. of n = 3 (apart from 0.25 nM docetaxel, n = 2) independent experiments. The total numbers of cells analysed in these experiments were as follows: DMSO: 1800; 0.25 nM docetaxel: 936; 0.5 nM docetaxel: 746. *p < 0.05 vs corresponding DMSO. (c) HeLa cells were transfected with a control siRNA (si control) or treated with transfection reagent alone (oligo). Cells were detached 24 h post transfection and plated on coverslips in 12 well plates. The following day, cells were treated with the indicated concentrations of docetaxel or vehicle alone (DMSO) and incubated for further 48 h before fixing and staining. Data indicate the number of cells containing n = 1, n = 2 or n ≥ 3 nuclei, expressed as percentage of total number of cells and are means ± s.e.m. of n = 3 independent experiments. The total numbers of cells analysed in these experiments were as follows: oligo DMSO: 1532; oligo 0.25 nM docetaxel: 1291; oligo 0.5 nM docetaxel: 809; si control DMSO: 1171; si control 0.25 nM docetaxel: 1170; si control 0.5 nM docetaxel: 719. *p < 0.05, **p < 0.01, ***p < 0.001 vs corresponding DMSO. [file 13046_2019_1472_MOESM8_ESM.pdf]

**a**

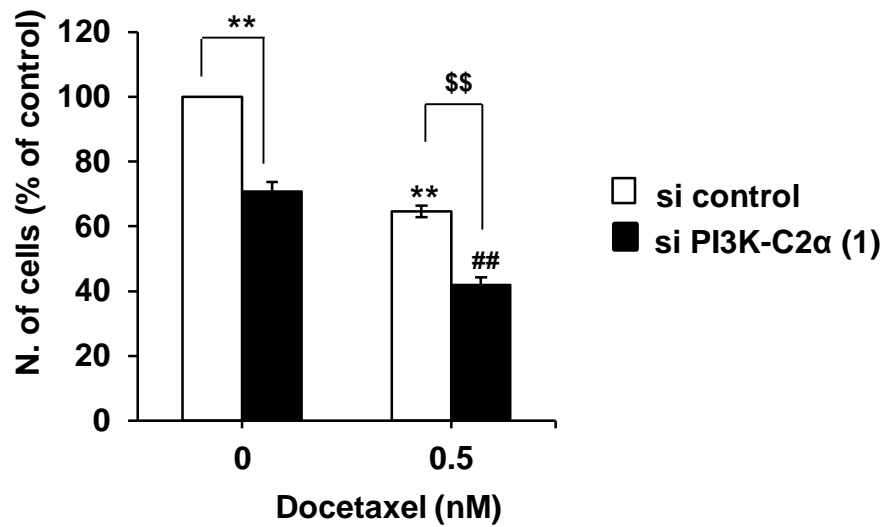

**b**

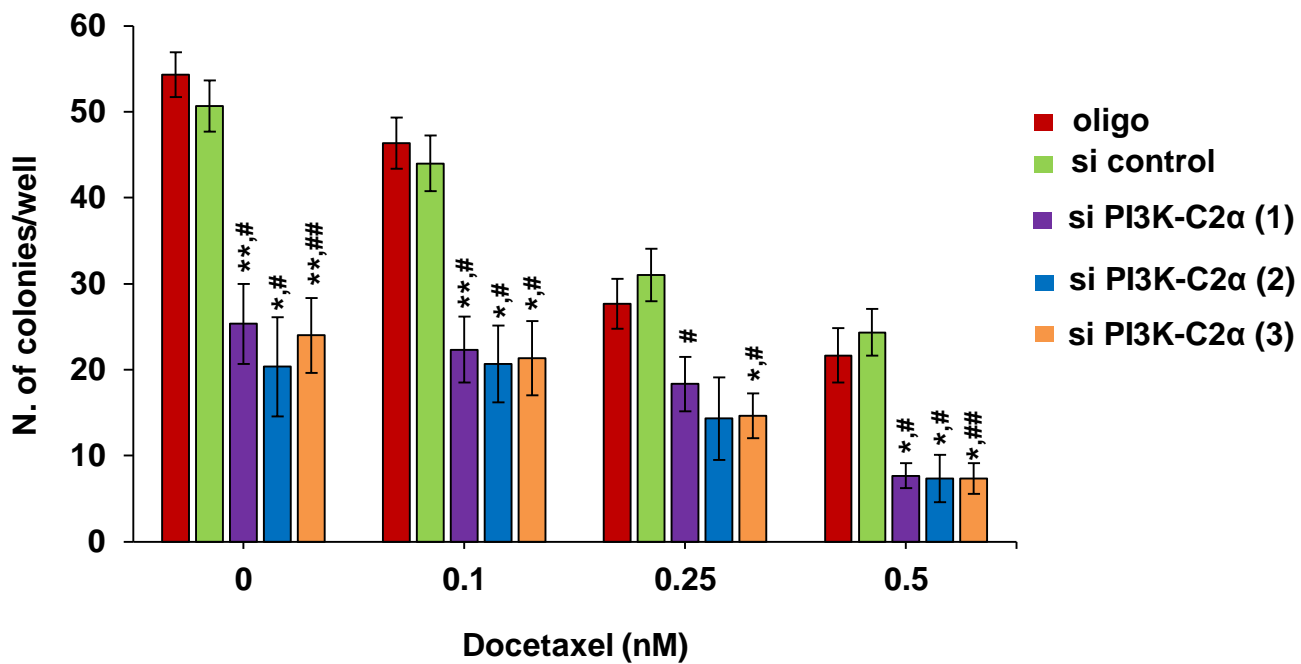

Supplement: Supplementary file 9 — Additional file 9: Figure S8. Combination of PI3K-C2α downregulation and docetaxel inhibits 2D colonies in clonogenic assays. (a) PC3 cells were transfected with a siRNA targeting PI3K-C2α or a non targeting siRNA. After 24 h, cells were incubated in complete media supplemented with 0.5 nM docetaxel or DMSO for further 48 h. The number of cells was assessed by cell counting. Data are expressed as percentage of cells transfected with si control and treated with DMSO and are means ± s.e.m. of n = 3 independent experiments performed in duplicate. **p < 0.01 vs si control/DMSO; ##p < 0.01 vs si PI3K-C2α/DMSO; $$p < 0.01 vs si control/docetaxel (two tailed, unpaired t-Test with Welch’s correction). (b) PC3 were transfected with the indicated siRNAs or transfection reagent alone (oligo) for 24 h before being detached and plated as single cells. Cells were incubated in complete media for 10 days in the presence of the indicated concentrations of docetaxel (or vehicle) before being fixed and stained with crystal violet. Data indicate the number of colonies (> 65 cells) and are means ± s.e.m. of n = 3 independent experiments performed in duplicate. *p < 0.05, **p < 0.01 vs corresponding oligo; #p < 0.05, ##p < 0.01 vs corresponding si control (two tailed, unpaired t-Test with Welch’s correction). [file 13046_2019_1472_MOESM9_ESM.pdf]

# Supplementary Figure S9

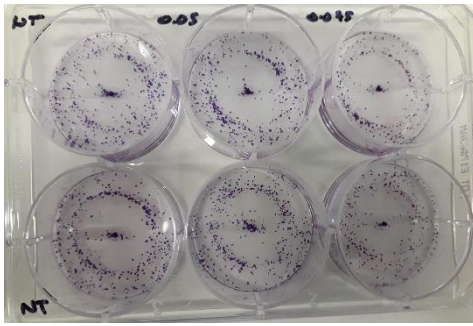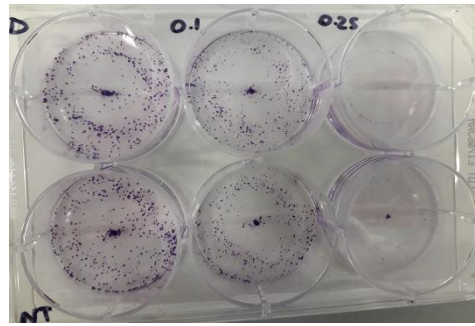

NT

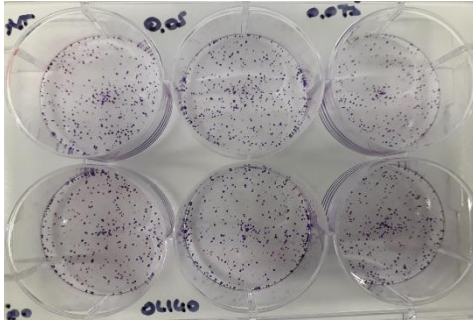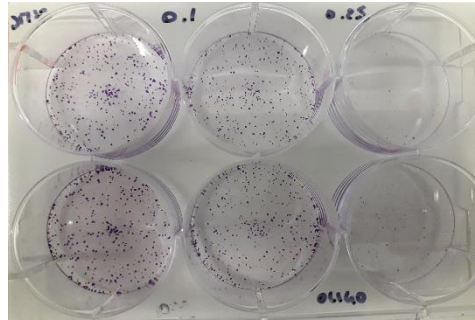

oligo

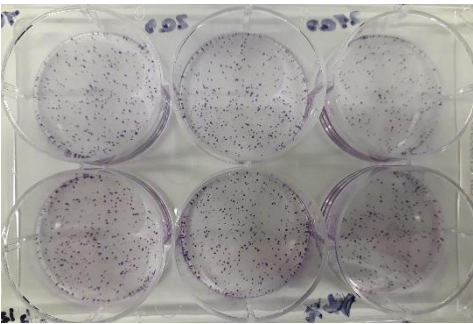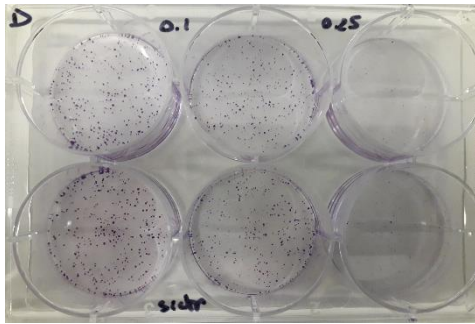

si control

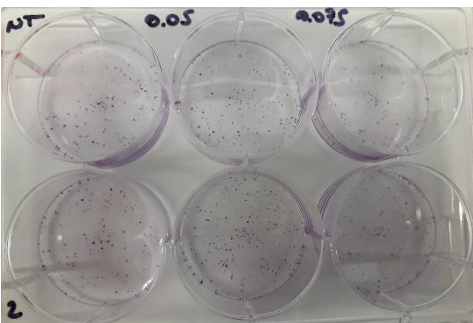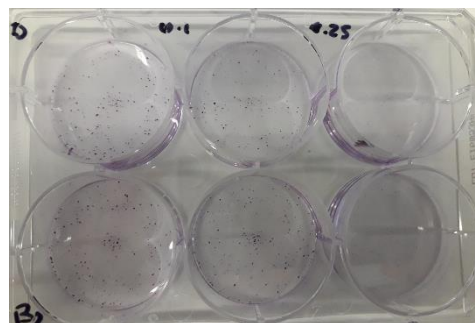

si PI3K-C2β(2)

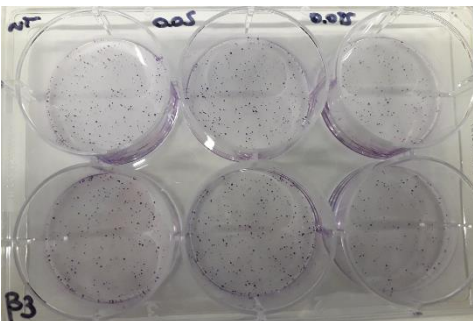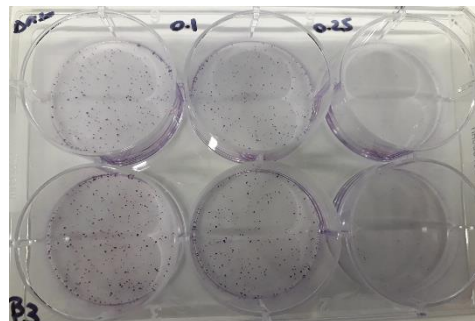

si PI3K-C2β(3)

Supplement: Supplementary file 10 — Additional file 10: Figure S9. Effect of combination of PI3K-C2β downregulation and docetaxel on 2D colonies of HeLa cells in clonogenic assays. HeLa cells were transfected with siRNAs targeting PI3K-C2β, a control siRNA (si control) or treated with transfection reagent alone (oligo). Not transfected (NT) cells were also used as additional control. Cells were detached 24 h post transfection and plated as single cells (100 or 200 or 400 cells/well) in 6 well plates in duplicate. Cells were incubated in complete media for 7 days in the absence (“NT”) or presence of the indicated concentrations of docetaxel (or vehicle, DMSO) before being fixed and stained with crystal violet. Representative images of 6 well plates are shown. [file 13046_2019_1472_MOESM10_ESM.pdf]

**a**

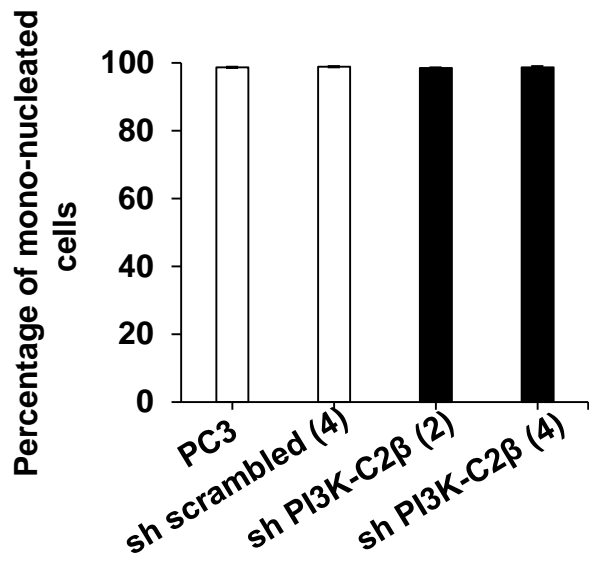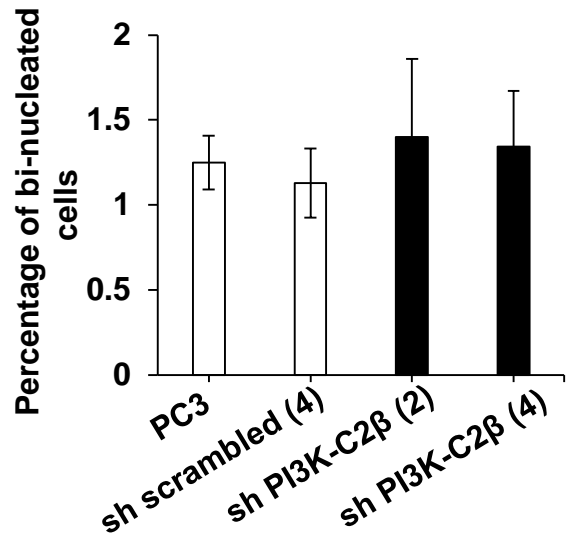

**b**

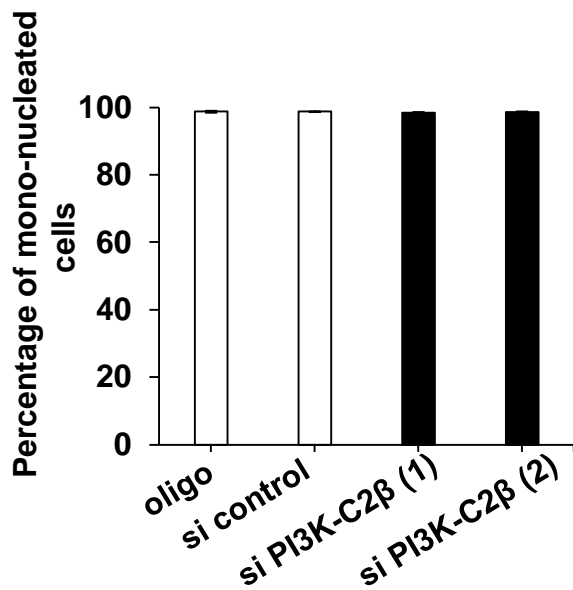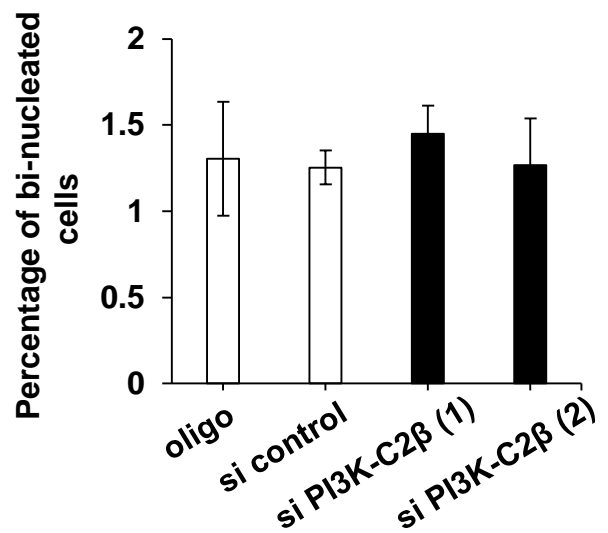

**c**

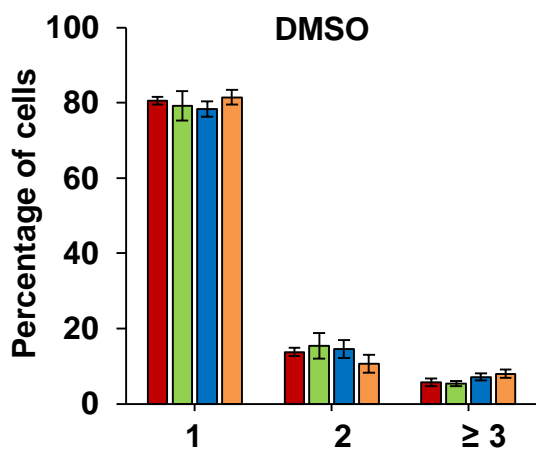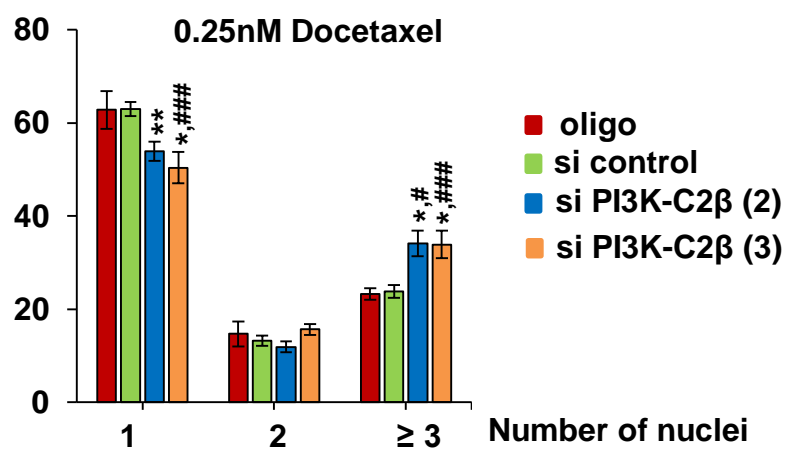

Supplement: Supplementary file 11 — Additional file 11: Figure S10. Effect of PI3K-C2β downregulation and docetaxel on multi-nucleation. (a, b) The indicated cells were treated as described in Fig. 6. Graphs indicate the number of cells treated with DMSO (in parallel to cells treated with docetaxel, presented in Fig. 6) and containing n = 1 and n = 2 nuclei. Data are expressed as percentage of total number of cells and are means ± s.e.m. of n = 6 (PC3 and sh scrambled), n = 4 [sh PI3K-C2β (3)] and n = 3 [sh PI3K-C2β (4)] (a) or n = 4 (b) independent experiments. In these conditions, no difference in the percentage of multi-nucleated cells was detected between the cell lines [PC3: 0.2±0.08; sh scrambled: 0.07±0.05; sh PI3K-C2β (3): 0.26±0.2; sh PI3K-C2β (4): 0.04±0.04] and no multi-nucleated cells were detected in cells transfected with transfection reagent or with si control. Multi-nucleation was detected only in one out of three experiments for si PI3K-C2β (1)-transfected cells [average percentage: 0.5±0.5, n = 3] and in two out of three experiments for cells transfected with si PI3K-C2β (2) [average percentage: 0.4±0.3, n = 3]. (c) HeLa cells were transfected as indicated, detached 24 h post transfection and plated on coverslips. The following day, cells were treated with 0.25nM docetaxel or DMSO and incubated for further 72 h before fixing and staining. Data indicate the number of cells containing n = 1, n = 2 or n≥3 nuclei, expressed as percentage of total number of cells and are means ± s.e.m. of n = 5 independent experiments [apart from si PI3K-C2β (3) DMSO, n = 4]. The total numbers of cells analysed in these experiments were as follows: oligo DMSO: 2582; oligo 0.25nM docetaxel: 1658; si control DMSO: 1658; si control 0.25nM docetaxel: 1122; si PI3K-C2β (2) DMSO: 1290; si PI3K-C2β (2) 0.25nM docetaxel: 711; si PI3K-C2β (3) DMSO: 846; si PI3K-C2β (3) 0.25nM docetaxel: 884. *p<0.05, **p<0.01, vs corresponding oligo; # p<0.05, ###p<0.001 vs corresponding si control. [file 13046_2019_1472_MOESM11_ESM.pdf]
